# Cell‐type‐specific gating of gene regulatory modules as a hallmark of early immune responses in Arabidopsis leaves

**DOI:** 10.1111/nph.70858

**Published:** 2026-01-07

**Authors:** Shanshan Wang, Ilja Bezrukov, Pin‐Jou Wu, Hannah Gauß, Marja Timmermans, Detlef Weigel

**Affiliations:** ^1^ Department of Molecular Biology Max Planck Institute for Biology Tübingen 72076 Tübingen Germany; ^2^ Center for Plant Molecular Biology University of Tübingen 72076 Tübingen Germany; ^3^ Institute for Bioinformatics and Medical Informatics University of Tübingen 72076 Tübingen Germany

**Keywords:** *Arabidopsis thaliana*, *cue1*, gene regulatory network (GRN), growth‐defense trade‐off, plant immunity, *Pseudomonas syringae*, single‐cell RNA‐seq

## Abstract

In plants, multiple cell types contribute to immunity, but what division of labor exists among cell types when immunity is activated? We compared, at single‐cell resolution, the response of *Arabidopsis thaliana* leaf cells during pattern‐triggered and effector‐triggered immunity (PTI/ETI), sampled at 3 and 5 h after infection with *Pseudomonas syringae* DC3000. Core defense modules were broadly shared across cell clusters, but their activation varied in timing and intensity, with key immune receptors also showing cell type–specific expression dynamics. Mesophyll cell populations could be distinguished based on their resilience patterns: after the initial response, some populations continue to express defense genes at high levels during both PTI and ETI, while others quickly reinitiate growth‐related gene expression programs but only during PTI. Gene regulatory network inference revealed WRKY‐regulated modules enriched in cells sensing effectors, while salicylic acid biosynthesis regulators were activated in complementary clusters. Analysis of *cue1* mutants demonstrated that core immune responses are robust to altered leaf architecture. In addition, we uncovered cryptic defense pathways, including sucrose‐responsive modules, in this mutant. By capturing early immune responses at high resolution, our study reveals cell type–specific coordination of plant immunity and provides a framework for decoding immune signaling networks.

**Table:** 

	Contents	
	Summary	2007
I.	Introduction	2007
II.	Materials and Methods	2009
III.	Results	2010
IV.	Discussion	2018
	Acknowledgements	2022
	References	2022

## I. Introduction

Plant immunity operates through two interconnected layers: pattern‐triggered immunity (PTI), initiated by cell‐surface pattern recognition receptors (PRRs) that sense conserved microbial patterns, and effector‐triggered immunity (ETI), a more robust response mediated by intracellular nucleotide‐binding domain and leucine‐rich repeat (NLR) proteins recognizing pathogen effectors (Jones & Dangl, 2006). While these layers are known to act synergistically and potentiate each other (Ngou *et al*., 2021; Yuan *et al*., 2021), how they are coordinated within the heterogeneous landscape of leaf tissues, especially at the earliest infection stages, remains incompletely understood.

Central to PTI are receptor‐like kinases (RLKs) and their close homologs, receptor‐like cytoplasmic kinases (RLCKs), which form a large and diverse family of membrane‐localized receptors that perceive pathogen‐associated molecular patterns (PAMPs) and initiate downstream signaling cascades (Shiu *et al*., 2004; Boutrot & Zipfel, 2017; Ngou *et al*., 2022). RLKs are composed of extracellular ligand‐binding, transmembrane, and intracellular kinase domains. RLCKs, which lack the extracellular and transmembrane regions, often functionally and physically associate with RLKs to regulate downstream signaling (Hailemariam *et al*., 2024). RLK‐RLCK modules thus often represent the first line of defense by detecting extracellular cues and triggering immune signaling. In parallel, NLR proteins can be grouped into coiled‐coil‐NLR (CNL), TIR‐NLR (TNL), *RPW8‐like* coiled‐coil‐NLR (RNL) based on the structure of N‐terminal domains, acting as intracellular immune sensors that can detect specific pathogen effectors, activating robust ETI responses often associated with localized cell death and systemic resistance (Jones *et al*., 2016; Duxbury *et al*., 2021). Recent studies have suggested that RLKs, RLCKs, and NLRs function synergistically to regulate PTI and ETI (Ngou *et al*., 2021; Yuan *et al*., 2021). Given their fundamental roles in pathogen perception and immune activation, understanding the spatial and temporal expression patterns of RLK‐RLCK modules and NLRs across diverse leaf cell types is critical to deciphering how plants coordinate effective immunity at the cellular level.

Bulk RNA sequencing studies have revealed distinct transcriptional dynamics in PTI and ETI: While PTI induces rapid, transient changes in gene expression, ETI elicits more robust and sustained responses (Tsuda & Katagiri, 2010; Mine *et al*., 2018; Bjornson *et al*., 2021). PTI and ETI share a large cohort of induced genes (Navarro *et al*., 2004), even though there are differences in downstream regulatory mechanisms. While salicylic acid (SA), jasmonic acid (JA), and ethylene act primarily synergistically during PTI, they do so in a more redundant manner during ETI (Hillmer *et al*., 2017; Mine *et al*., 2018). Recent studies have also demonstrated mutual potentiation between PTI and ETI, in which each pathway amplifies the other's transcriptional output, with the PTI/ETI cross talk enhancing defense durability (Ngou *et al*., 2021; Yuan *et al*., 2021).

The *Arabidopsis thaliana* (Arabidopsis) leaf is a mosaic of specialized cell types, with the major types being mesophyll, epidermal, guard, and vascular cells (Liu *et al*., 2020; Kim *et al*., 2021; Lopez‐Anido *et al*., 2021). In addition, there are many transient cell states shaped by developmental gradients, metabolic activity, and microenvironmental cues (Lopez‐Anido *et al*., 2021; Procko *et al*., 2022; Tenorio Berrío *et al*., 2022; Guo *et al*., 2025). Recent spatial transcriptomic and single‐cell analyses have revealed that even within a single tissue type, such as the mesophyll, cells exhibit region‐specific functional specialization. While photosynthesis is the primary function of mesophyll cells, gene expression profiling demonstrates that photosynthetic efficiency and related processes vary along the medio‐lateral axis of the leaf (Xia *et al*., 2022). Leaf cells differ markedly in their developmental age and metabolic status, with senescence and nutrient allocation occurring in a highly coordinated yet cell‐type‐specific manner across the Arabidopsis leaf, as demonstrated with single nucleus expression data (Guo *et al*., 2025). A comprehensive analysis of transcriptional profiles in time and space has highlighted the remarkable complexity of cell types and states during the Arabidopsis life cycle (Lee *et al*., 2025). Because of this extensive cellular and functional heterogeneity, leaves are poised to respond to pathogen challenges not uniformly, but with finely tuned, cell‐type, and context‐dependent immune programs.

Recent advances in single‐cell transcriptomic and epigenomic technologies have revolutionized our understanding of plant immunity, revealing in considerable detail temporal and spatial heterogeneity in immune responses. For example, single‐cell RNA‐seq (scRNA‐seq) analyses of Arabidopsis plants infected with *Pseudomonas* highlighted both cell specialization and coordination of immune responses between cell types (Delannoy *et al*., 2023), as well as immune active cell states in mesophyll cells with elevated expression of *FLG22‐INDUCED RECEPTOR‐LIKE KINASE 1* (*FRK1*), *CALMODULIN‐BINDING PROTEIN 60 G* (*CBP60g*), and LIPOPROTEIN 1 (*LipoP1*), and particularly susceptible cell states marked by *EXPANSIN A10* (*EXPA10*) and aquaporin genes (Zhu *et al*., 2023). Tang *et al*. (2023), working with Arabidopsis and the fungal pathogen *Colletotrichum higginsianum*, found vasculature‐specific enrichment of NLR expression and guard cell‐specific regulation of abscisic acid (ABA) signaling, which mediates stomatal closure upon direct fungal contact. Single‐nuclei RNA‐seq (snRNA‐seq) combined with single‐cell ATAC‐seq (assay for transposase‐accessible chromatin using sequencing) revealed rare PRIMER cells as immune signaling hubs, with high expression of *BONZAI 3* (*BON3*) and genes whose promoters are enriched for CAMTA and GT‐3A motifs, while adjacent bystander cells upregulate systemic resistance genes such as *AGD2‐LIKE DEFENSE RESPONSE PROTEIN 1* (*ALD1*) and *FLAVIN‐CONTAINING MONOOXYGENASE 1* (*FMO1*) (Nobori, 2025; Nobori *et al*., 2025).

Here, we present a single‐cell transcriptomic atlas of Arabidopsis wild‐type (WT) and mutant leaves challenged with either virulent (‘empty vector’, EV) or avirulent (expressing AvRpt2) *Pseudomonas syringae* pv *tomato* (*Pst*) DC3000. DC3000 suppresses plant immunity using a suite of effector proteins and toxins, but when it also expresses the AvrRpt2 effector, which is recognized by the NLR receptor RESISTANCE TO P. SYRINGAE 2 (RPS2), plants mount an ETI response (Whalen *et al*., 1991; Kunkel *et al*., 1993; Mackey *et al*., 2003). We show that early immune responses are driven by a set of shared co‐expression modules whose activation is gated at the level of cell types. Key regulons, controlled by TRIHELIX DNA‐BINDING FACTOR (GT FACTOR) BINDING 3A (GT‐3A), and SA responsive or biosynthesis‐related transcription factors (TFs) coordinate these modules within distinct mesophyll clusters. We also identify two contrasting mesophyll populations: one that always prioritizes defense over growth and another that does the opposite – but only during PTI. Moreover, we demonstrate that receptor expression dynamics further sculpt a heterogeneous yet robust immune response landscape. Finally, by profiling *cue1*‐6 mutants, which have highly altered leaf architecture and impaired shikimate biosynthesis (Li *et al*., 1995; Streatfield *et al*., 1999; Voll *et al*., 2003; Pérez‐Pérez *et al*., 2013), we demonstrate the robustness of immune modules in the face of major shifts in cell‐type populations in leaves and reveal a role for sucrose‐derived pathways in bolstering early defense.

## II. Materials and Methods

### 1. Reagents and kits

The following reagents and kits were used: Chromium Next GEM Single Cell 3′ Kit v.3.1 (10× Genomics, PN‐1000268), Chromium Next GEM Chip G Single Cell Kit (10× Genomics, PN‐1000120), TWEEN 20 (P1379; Sigma‐Aldrich), SPRIselect Bead‐Based Reagent (B23317; Beckman Coulter, Brea, CA, USA), Low TE Buffer (10 mM Tris–HCl pH 8.0, 0.1 mM EDTA) (J75793.AP; Thermo Fisher Scientific, Waltham, MA, USA), glycerol (G5516; Sigma‐Aldrich), RNeasy Plant Mini Kit (74904; Qiagen), diethiothreitol (DTT) (R0861; Thermo Fisher Scientific), DNase I, RNase‐free (EN0521; Thermo Fisher Scientific), diethyl pyrocarbonate (DEPC) (D5758; Sigma‐Aldrich), RNase inhibitor/RNaseOUT (10777019; Invitrogen), MES hydrate (69890; Sigma‐Aldrich), d‐mannitol (M1902; Sigma‐Aldrich), calcium chloride (A119.1; Roth, Karlsruhe, Germany), potassium chloride (6781.1; Roth), magnesium chloride hexahydrate (2189.1; Roth), sodium chloride (3957.1; Roth), Cellulase‐RS (C8003; Duchefa Biochemie, Haarlem, the Netherlands), and Macerozyme‐R10 (M8002; Duchefa Biochemie).

### 2. Plant material and growth conditions

Seeds of the *Arabidopsis thaliana* reference accession Col‐0 and the *cue1*‐6 mutant (Streatfield *et al*., 1999) in the same background (kindly provided by Dr R. E. Häusler, University of Cologne) were surface‐sterilized with 30% commercial bleach and stratified at 4°C for 1 wk before sowing on soil. Seven‐ to 10‐d‐old seedlings were transplanted into individual pots and grown at 8 h : 16 h, 23°C, light : dark. Unless otherwise noted, Col‐0 plants were 5–6 wk and *cue1*‐6 plants 9 wk.

### 3. Bacterial infections and bacterial growth measurements

*Pseudomonas syringae* pv tomato (*Pst*) DC3000 (EV) and *Pst* DC3000 (AvrRpt2) were cultured overnight at 28°C in Luria Broth (LB) medium with kanamycin (50 mg l^−1^). Cells were pelleted by centrifugation, washed, and resuspended in 10 mM MgCl_2_. For RNA‐seq samples, suspensions were adjusted to OD_600_ = 0.005; for bacterial growth curves, to OD_600_ = 0.0001 to minimize hypersensitive cell death. The abaxial surfaces of leaves were infiltrated with a needle‐less syringe, blotted dry, and harvested at 3 h post infection (hpi) and 5 hpi for scRNA‐seq samples, at 4 hpi for bulk RNA‐seq samples, or at 0 d post infection (dpi) and 3 dpi for bacterial growth measurements. Each treatment comprised three biological replicates (2–3 leaves per Col‐0 plant and 3–6 leaves per *cue1*‐6 plant).

For measurements of bacterial growth, leaf disks were harvested with a 6‐mm‐diameter punch on Day 0 (immediately after infiltration) and Day 3. For Day 0 samples, six leaves from two plants were pooled (two disks per leaf), and four disks were combined as one sample. For Day 3, four disks from one plant (two disks per leaf) constituted a sample. Disks were ground in 200 μl infiltration buffer, serially diluted (10×, 100×, 1000×), and 10 μl of each dilution was spotted on LB agar plates containing kanamycin (50 μg ml^−1^). Plates were incubated at 28°C for 48–72 h, and bacterial growth was quantified as colony‐forming units per leaf area (CFU cm^−2^).

### 4. Protoplast isolation

Protoplasts were isolated as described, with sucrose centrifugation to remove debris (Yoo *et al*., 2007; Jeong *et al*., 2021). Leaves were incubated in digestion buffer (1.5% Cellulase‐RS, 0.3% Macerozyme R10, 20 mM MES pH 5.7, 0.6 M mannitol, 20 mM KCl, 10 mM CaCl_2_, 1% BSA) for 1 h at room temperature with gentle agitation. After low‐speed centrifugation (150 ***g***, 5 min, 4°C), the pellet was resuspended in MMC solution (10 mM MES, 0.47 M mannitol, 10 mM CaCl_2_) and layered over 6 ml of 6 M sucrose. Following centrifugation (80 ***g***, 9 min), the middle layer was recovered, washed in 0.5 M mannitol, and pelleted (150 ***g***, 5 min, 4°C). Protoplasts were resuspended in a solution with 0.25 M mannitol and 3 mM sucrose for scRNA‐seq. For bulk RNA‐seq, cells were collected before sucrose gradient centrifugation.

### 5. Bulk RNA‐seq and analysis

Total RNA was extracted from frozen protoplast pellets using the RNeasy Plant Mini Kit (Qiagen). Libraries were prepared from 500 ng RNA as described (Cambiagno *et al*., 2021) and sequenced on an Illumina NextSeq 2000 instrument with 100‐bp single‐end reads. Reads were aligned to the TAIR10 genome (Araport11 GTF) (Cheng *et al*., 2017) using the nf‐core/rna‐seq pipeline (v.2.0.0) with default parameters and STAR‐RSEM for alignment and quantification. Gene‐level counts were processed in DESeq2 v.1.46.0 (Love *et al*., 2014). Genes with total counts ≤ 5 were removed, data were rlog‐normalized, and differentially expressed genes (DEGs) were identified with a Wald test (false discovery rate (FDR) < 0.05, |log_2_ FC| ≥ 1). *P*‐values were adjusted for multiple testing using the Benjamini–Hochberg procedure.

### 6. ScRNA‐seq and processing

Protoplasts were counted by light microscope and loaded onto the 10× Chromium Controller using Next GEM 3′ v.3.1 reagents. Libraries were sequenced on a NextSeq 2000 instrument (90 bp read 2, dual index). Reads were processed with the nf‐core/scrnaseq pipeline v.2.0.0 (Cell Ranger v.7.0.0) against the Arabidopsis TAIR10 reference.

Raw UMI matrices were imported into Seurat v.4 (Hao *et al*., 2021). Only cells with nCount_RNA > 1000, 500 < nFeature_RNA < 8000 and chloroplast transcripts < 15% were retained. To identify and remove putative doublets, we used DoubletFinder v.2.0.3 (McGinnis *et al*., 2019).

### 7. ScRNA‐seq data integration and clustering

Raw counts were first normalized and variance‐stabilized using Seurat's SCTransform (Hafemeister & Satija, 2019). To integrate across batches and conditions, we used Seurat's anchor‐based reciprocal PCA (RPCA) workflow, followed by IntegrateData to produce an ‘integrated’ assay. Clustering analysis was performed with RunPCA (npcs = 50), followed by FindNeighbors (). Cell clusters were identified with FindClusters () at a resolution of 0.8. Uniform manifold approximation and projection (UMAP) visualization was generated from the Harmony embeddings (reduction = ‘harmony’, dims = 1 : 30). We ran RunUMAP() once on the Harmony embeddings and used the resulting UMAP coordinates for all figures. The resulting Seurat object (seurat.obj including umap_1 and umap_2) is uploaded and available at doi: 10.5281/zenodo.16533110.

### 8. Identification of DEGs in scRNA‐seq data and gene module clustering

For each cell cluster, DEGs between mock and infection treatment of the same genotype (Col‐0 or *cue1*‐6) were identified with FindMarkers on SCT assay. All DEGs (adj *P* < 0.05, |log_2_ FC| > 0.75) were combined and partitioned into six co‐expression modules by *k*‐means clustering (ComplexHeatmap) (Gu *et al*., 2016), using Euclidean distance for Fig. 2(c)/Supporting Information Fig. S4(a), Fig. 3(c)/Fig. S7(a), and Fig. 6(f)/Fig. S13, and *k* = 4, 3,4 for Fig. S9(a–c). For the heatmaps of RLK, RLCK, and NLR gene expression, gene‐level variance was calculated across all samples, and the bottom decile (including genes with zero or near‐zero expression in all conditions) was excluded. This conservative threshold was used to preserve condition‐specific signals while reducing noise.

### 9. Gene regulatory network inference

MINI‐EX (Ferrari *et al*., 2022) was run as described (Cao *et al*., 2023) on individual Seurat objects and corresponding DEG lists for each cell cluster. Regulons were ranked by ‘borda_clusterRank’ specificity, and the top 10 regulons in each cell cluster were visualized with presence/absence heatmaps. Note that there are two cell clusters each for epidermis and guard cells (epidermis_1 and _2, guard cell_1 and _2).

### 10. Statistical analysis of RLK, RLCK, NLR genes expression scores

To assess changes in RLK, RLCK, and NLR genes expression scores in *Pst* DC3000 (EV) and *Pst* DC3000 (AvrRpt2) infected samples within each cell cluster, we performed pairwise comparisons between treatments using the Wilcoxon rank‐sum test. Analyses were conducted independently for each cell cluster using the rstatix R package (v.0.7.2). To account for multiple hypothesis testing, *P*‐values were adjusted using the Benjamini–Hochberg (BH) method to control the FDR. A *P*‐adj value < 0.05 was considered statistically significant.

## III. Results

### 1. A single‐cell RNA‐seq atlas of early responses to virulent and avirulent *Pseudomonas* infection

**Fig. 1:**
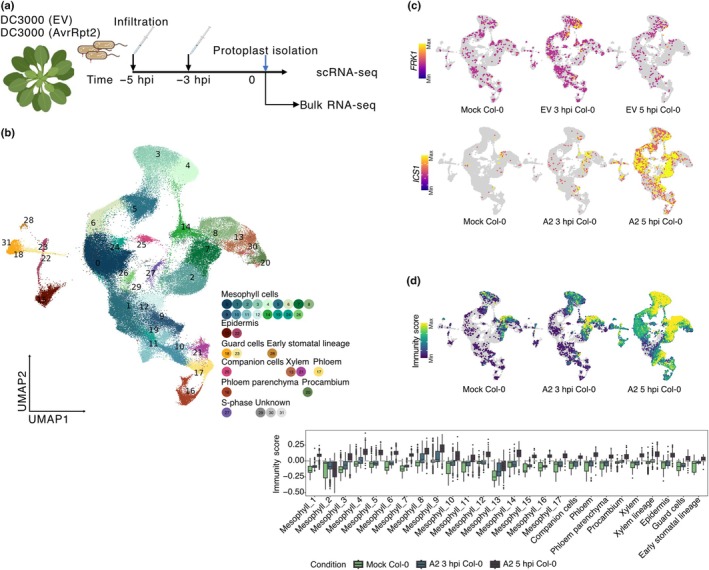
Single‐cell RNA‐seq atlas of *Arabidopsis thaliana* leaves during early *Pseudomonas syringae* infection. (a) Experimental design. Fourteen‐day‐old rosettes were syringe‐infiltrated with infiltration buffer only (mock), *P. syringae* pv tomato (*Pst*) DC3000 (empty vector, EV), or *Pst* DC3000 (AvrRpt2) at OD_600_ = 0.005. Leaves were sampled 3 and 5 h post infection (hpi). Protoplasts were immediately isolated for single‐cell RNA‐seq (scRNA‐seq) and bulk RNA‐seq. (b) Uniform manifold approximation and projection (UMAP) of transcriptomes from 109 192 cells, colored and numbered by Seurat cluster. Clusters were assigned to major cell types with known marker genes: mesophyll, epidermis (including guard‐cell lineage), companion cells, phloem (parenchyma and procambium), xylem, and an S‐phase cluster. (c) Layered UMAP plots showing per‐cell expression of the defense marker genes *FRK1* (upper) and *ICS1* (lower). Color scale denotes relative normalized expression. (d) Upper panel: UMAPs of the ‘Immunity Score’, computed with AddModule function in Seurat from bulk‐RNA‐derived differentially expressed genes (DEGs) in mock (green), A2 3 hpi (light teal), and A2 5 hpi samples (dark teal). Lower panel: boxplot showing the distribution of Immunity Score in each cell cluster, comparing mock, A2 3 hpi, and A2 5 hpi samples. For the boxplots, Center lines indicate medians; boxes span the IQR; whiskers extend to 1.5 × IQR. Empty vector (EV) and A2 are short for *Pst* DC3000 (EV) and *Pst* DC3000 (AvrRpt2), respectively.

We generated a time series of scRNA‐seq data for leaves of the Arabidopsis reference accession Col‐0 and the *cue1*‐6 mutant infiltrated with either virulent *Pst* DC3000 (EV) or avirulent *Pst* DC3000 (AvrRpt2), and MgCl_2_ for mock samples (Fig. 1a). Leaves were syringe‐infiltrated at a low dose of OD_600_ = 0.005. At this dose, only some cells are expected to have direct contact with the pathogen during early stages of infection (Delannoy *et al*., 2023; Zhu *et al*., 2023; Nobori *et al*., 2025). We harvested the samples at 3 and 5 hpi to capture the earliest transcriptional changes. To minimize circadian effects, isolation of protoplasts and subsequent 10× Genomics droplet scRNA‐seq preparation were always performed at the same time of day; infiltrations therefore were done at −5, −3, or 0 h relative to harvest and sample processing.

After quality filtering, we retained data from 109 192 cells from 15 samples (mean ≈ 6775 unique molecular identifiers and 2150 detected genes per cell; see Section II; Fig. S1). Clustering and marker‐based annotation with Seurat (Hao *et al*., 2021, 2024) identified 29 cell clusters that we could assign to the major histological cell types in the leaf: mesophyll (17 clusters), which constituted as expected the majority of cells; vascular tissue, including xylem (2 clusters), companion cells, phloem, phloem parenchyma, and procambium; epidermis (2 clusters), including nonguard cells (called ‘epidermis’ from here on) along with guard cells (2 clusters), and cells belonging to the early stomatal lineage, as well as cells in S‐phase (total 29 clusters) and three small unassigned populations lacking canonical cell‐type markers (Figs 1b, S2; Tables S1, S2).

We first examined two broadly used sentinel markers, *FRK1*, diagnostic of a transient early response during PTI (Asai *et al*., 2002), and *ISOCHORISMATE SYNTHASE 1* (*ICS1*), a hallmark of SA synthesis upon pathogen attack (Wildermuth *et al*., 2001). Upon *Pst* DC3000 (EV) challenge, *FRK1* expression peaks at 3 hpi and subsides by 5 hpi, reflecting a defense spike, consistent with a previous study using bulk RNA sequencing (Lewis *et al*., 2015). Under *Pst* DC3000 (AvrRpt2) challenge, *ICS1* expression increases steadily until 5 hpi, indicating prolonged SA biosynthesis activation (Fig. 1c).

To generate a set of immune response markers as well as to rule out transcriptomic changes due to protoplasting, we performed bulk RNA sequencing with protoplasts isolated after infection. We identified 897 DEGs (|log_2_FC| ≥ 1, FDR < 0.05; 565 up, 332 down; Table S3) from bulk RNA‐seq of protoplasts 4 hpi after infection with *Pst* DC3000 (AvrRpt2) when compared with protoplasts from mock samples. Note that comparison of *Pst* DC3000 (EV) to the mock sample yielded only 61 DEGs (Table S3).

We determined an integrated Immunity Score based on the 897 DEGs by calculating the sum of relative expression of all DEGs. For each cell in our *Pst* DC3000 (AvrRpt2) infected samples, we could thereby map the net transcriptional shift toward activation or repression of immunity (Figs 1d, S3). The Immunity Score demonstrated that all cell clusters have the ability to activate an immune response. The Immunity Score was highest in Mesophyll_9 and Mesophyll_8 at 3 hpi, marking these as early response cell clusters.

### 2. Cluster‐modulated deployment of defense and housekeeping modules under virulent *Pst* DC3000 (EV) challenge

**Fig. 2:**
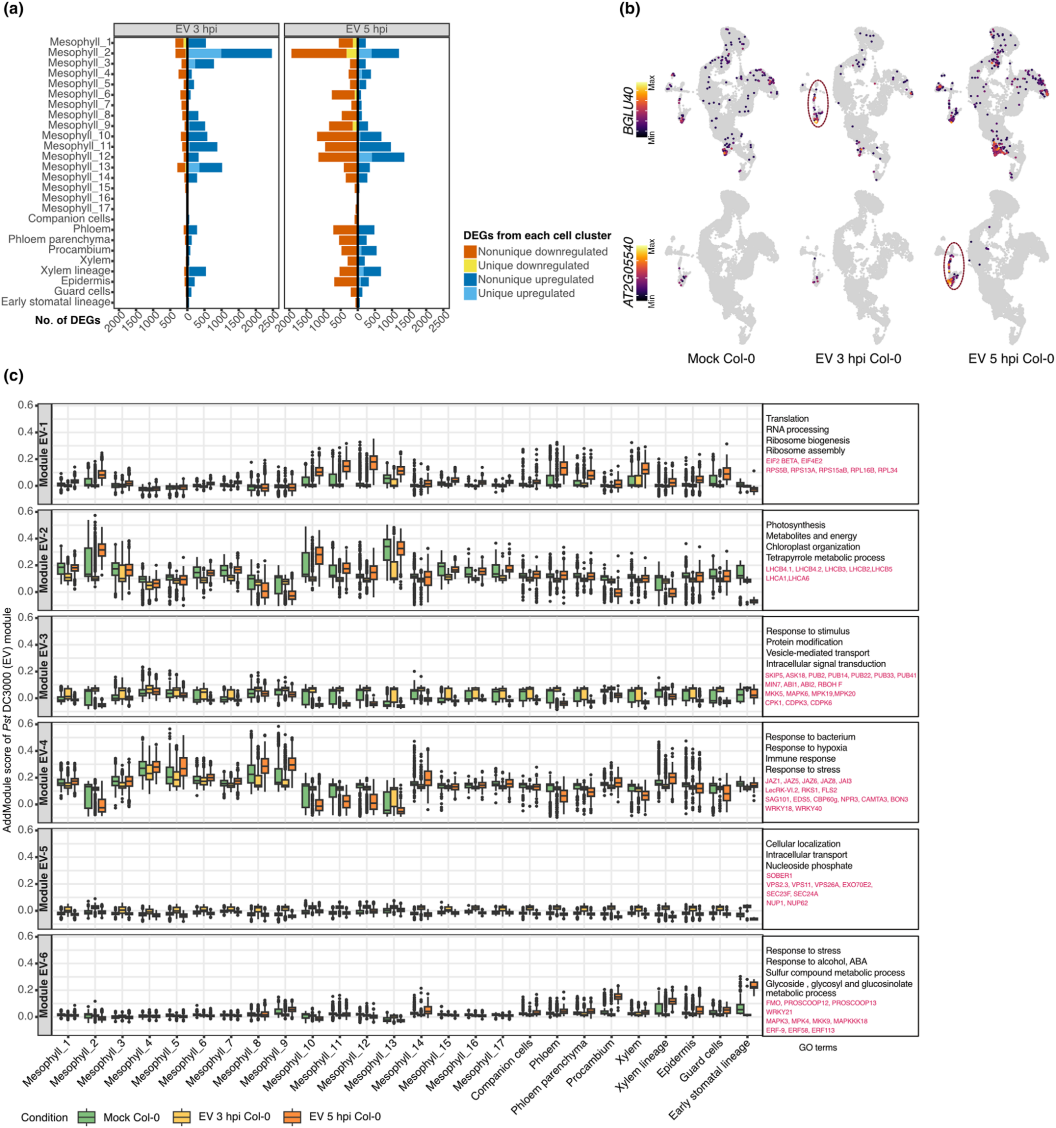
Early dynamic defense response of *Arabidopsis thaliana* leaf cell clusters upon *Pst* DC3000 (empty vector (EV)) infection. (a) Bar plots of differentially expressed genes (DEGs) (*P*‐adj < 0.05, |log_2_FC| > 0.75) in each Seurat‐defined cell cluster in EV 3 h post infection (hpi) vs mock (left) and EV 5 hpi vs mock (right) samples. Bars are stacked by DEG category as indicated on the right. Clusters are ordered by similarity. (b) Uniform manifold approximation and projection (UMAP) plots of two cluster‐specific marker genes, *BGLU40*/*AT1G* *26560* (upper row) and *AT2G05540* (lower row), in mock, EV 3 hpi, and EV 5 hpi samples. Color intensity indicates relative normalized expression. Red ovals highlight the clusters in which each gene is most strongly induced upon infection. (c) Boxplots of module scores for six co‐expression modules, calculated with Seurat's AddModuleScore using DEGs from all cell clusters comparing EV 3 hpi and EV 5 hpi with mock samples (as shown in (a)). The average of module scores per cell cluster is indicated. Center lines indicate medians; boxes span the IQR; whiskers extend to 1.5 × IQR. Color code of samples is given on the bottom. Top enriched GO terms for each module are listed to the right of each panel. Related genes are highlighted in red. EV, *Pseudomonas syringae* pv tomato (*Pst*) DC3000. GO, gene ontology.

To dissect how different types of genes are modulated across different cell populations during virulent challenge, we identified DEGs in each cell cluster (|log_2_FC| > 0.75, *P*‐adj < 0.05). In *Pst* DC3000 (EV)‐infected leaves, most, but not all, DEGs were shared across multiple clusters (Fig. 2a; Table S4). Examples of notable genes upregulated specifically after *Pst* DC3000 (EV) infection (Fig. 2b) were the known defense‐related gene for β‐GLUCOSIDASE 40 (*BGLU40*) (Xu *et al*., 2012; Yamada *et al*., 2020), and *AT2G05540*, a gene for a glycine‐rich protein that had not been linked to defense before (Suh *et al*., 2005). These genes showed distinct temporal and spatial patterns of induction, with *BGLU40* upregulated in the epidermis at 3 hpi and *AT2G05540* at 5 hpi.

To resolve coregulated functional modules, we performed *k*‐means clustering (Gu *et al*., 2016) on all DEGs identified in *Pst* DC3000 (EV)‐infected samples (3 hpi vs mock and 5 hpi vs mock comparisons for each cell cluster), partitioning genes into six distinct modules (Module EV‐1 to EV‐6) based on their expression profiles across cell clusters and time points (Figs 2c, S4; Table S5). Following infection with *Pst* DC3000 (EV), Module EV‐2, dominated by photosynthesis and chloroplast‐related genes, was transiently downregulated at 3 hpi before rebounding at 5 hpi in most of the cell clusters, likely reflecting a brief suspension of growth. On the contrary, Modules EV‐3 and EV‐5, which were enriched for housekeeping Gene Ontology (GO) terms, such as protein modification, vesicle‐mediated transport, and nucleoside phosphate, were transiently induced at 3 hpi across most clusters but returned to pre‐infection levels by 5 hpi. The defense‐related Module EV‐4 was downregulated at 3 hpi, but its expression recovered or even became elevated in most mesophyll cells at 5 hpi. By contrast, Module EV‐4 is upregulated in Mesophyll_2, _10, _11 and _13 at 3 hpi but becomes downregulated in these clusters at 5 hpi. Finally, Module EV‐6, which contains ABA signaling and glycoside/glucosinolate metabolism genes, was exclusively activated at 5 hpi in vascular, guard cells, and early stomatal‐lineage cells, pointing to a cell‐type specific hormone‐mediated stress response (Fig. 2c).

To identify the transcriptional drivers underlying the responses of the different clusters, we inferred gene regulatory networks (GRNs) defined by specific TFs with MINI‐EX (Ferrari *et al*., 2022). Regulons controlled by the relevant TFs were ranked by their cell‐cluster specificity (borda_clusterRank), and the top 10 regulons for each cluster for the mock, *Pst* DC3000 (EV) 3 hpi and *Pst* DC3000 (EV) 5 hpi samples were extracted and then visualized (Fig. S5; Tables S6–S8).

There were not only regulons that were shared across cell types and different infections but also unique regulons determined by cell type or the response to pathogen infection. In the mock condition, the networks regulated by RESPONSIVE TO HIGH LIGHT 41(RHL41/ZAT12), ABA REPRESSOR 1 (ABR1), MYB74, MYB102, MYB15, and ZINC FINGER OF ARABIDOPSIS THALIANA 6 (ZAT6) consistently appeared as top‐ranked regulons (Fig. S5). These six TFs are well‐known for their roles in abiotic or biotic‐stress responses and growth‐related pathways. For example, RHL41 regulates high‐light acclimation as well as cold and oxidative stress responses; ABR1, an ABA repressor, interacts with multiple *P. syringae* effectors; MYB74 controls osmotic stress to decrease plant growth; and MYB102 increases aphid susceptibility (Iida *et al*., 2000; Zhu *et al*., 2018; Bäumler *et al*., 2019; Schreiber *et al*., 2021; Ortiz‐García *et al*., 2022). Notably, their regulons were specifically enriched in the Mesophyll_2, _10, and _13 clusters (Fig. S5), indicating that this set of stress regulators helps define a broader GRN in uninfected tissue. Upon DC3000 (EV) infection, many of these same regulons remain active, but additional, cell‐type specific regulons emerge. For example, MYB96 (Seo *et al*., 2011), a key regulator of wax biosynthesis, defines a top regulon in epidermal clusters at 5 hpi (Fig. S5), implicating cuticular reinforcement in epidermal defense.

Taken together, we observed that shared defense and housekeeping gene modules are deployed broadly but that the onset and intensity of activation of each module are tuned at the cell‐cluster level, revealing a gating mechanism by which individual cell populations calibrate their response to pathogen attack. By reconstructing GRNs, we reveal a dual logic whereby common regulons maintain cluster identity while distinct, cell‐type‐specific networks fine‐tune each cluster's transcriptional response to pathogen challenge.

### 3. Cell cluster‐dependent deployment of defense and housekeeping modules under avirulent *Pst* DC3000 (AvrRpt2) infection

**Fig. 3:**
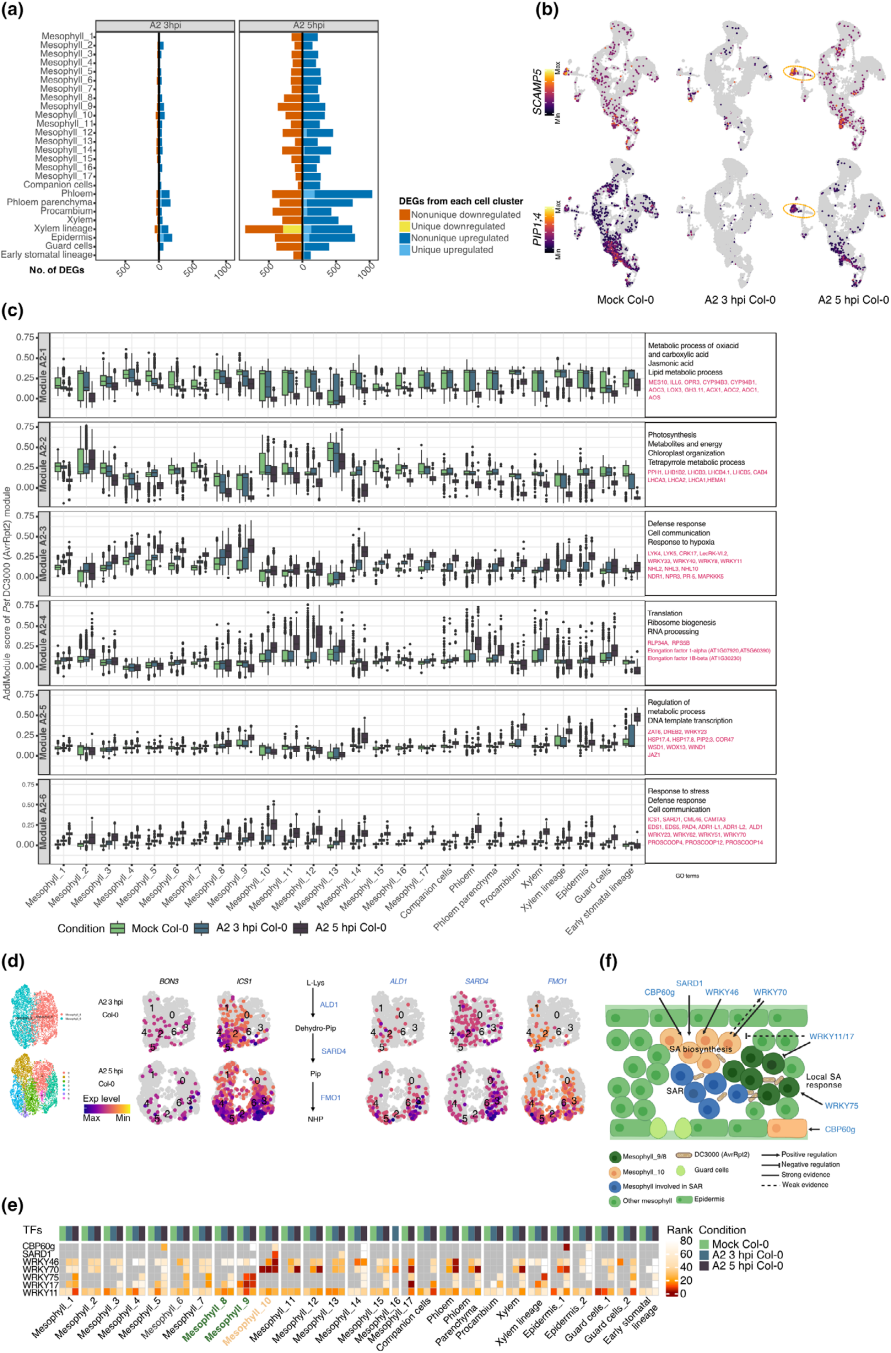
Early responses of *Arabidopsis thaliana* leaf cell clusters upon *Pst* DC3000 (AvrRpt2) infection. (a) Bar plots of differentially expressed genes (DEGs) (*P*‐adj < 0.05, |log_2_FC| > 0.75) in each cell cluster in A2 3 h post infection (hpi) vs mock (left) and A2 5 hpi vs mock (right) samples. Bars are stacked by DEG category as indicated on the right. (b) Uniform manifold approximation and projection (UMAP) plots of two cluster‐specific marker genes, *SCAMP5*/*AT1G32050* (upper row) and *PIP1;4*/*AT4G00430* (lower row) in mock, A2 3 hpi and A2 5 hpi samples. Colors denote relative normalized expression values. Red ovals highlight the clusters in which each gene is most strongly induced upon infection. (c) Boxplots of module scores for six co‐expression modules, calculated with Seurat's AddModuleScore using DEGs from all cell clusters in A2 3 hpi/5 hpi vs mock samples (as shown in (a)). The averages of module scores per cell cluster are indicated. Center lines indicate medians; boxes span the IQR; whiskers extend to 1.5 × IQR. Color code of samples is given on the bottom. Top enriched GO terms for each module are listed to the right of each panel, and related genes are highlighted in red. (d) Subclusters generated from Mesophyll_8 and _9 clusters (left), and the expression of immune‐related genes in these subclusters (right). (e) Heatmap of regulon enrichment ranks for seven salicylic acid (SA)‐pathway transcription factors across all clusters and conditions. The color of each tile (from white to dark red) indicates the relative rank (1 = strongest enrichment; 80 = weakest) of the predicted activity of a given transcription factor gene in a given cluster in mock (green), A2 3 hpi (light teal), and A2 5 hpi (dark teal) samples. Mesophyll_8, _9, and _10 are annotated in bold. (f) Schematic of *Arabidopsis thaliana* leaf cells showing SA biosynthesis in Mesophyll_10 and epidermis cells (orange), local SA response in Mesophyll_8 and _9 cells (dark green), and mesophyll cells that are involved in systemic acquired resistance (SAR) (blue). Key regulons controlled by WRKY75, WRKY11/17, SASRD1, CBP60g, WRKY46, WRKY70 are listed. Solid arrows indicate positive regulation, blunt bars negative regulation, and dashed lines weaker or indirect evidence. Note that in Mesophyll_8, _9, and _10, *ICS1* is upregulated (Fig. 1b,c; Supporting Information Table S9). Created with BioRender. Wang, S. (2025) https://BioRender.com/hbw8gz6. A2 is short for *Pseudomonas syringae* pv tomato (*Pst*) DC3000 (AvrRpt2). GO, gene ontology.

Upon *Pst* DC3000 (AvrRpt2) challenge, the majority of DEGs were shared across clusters, and only a small fraction was truly cluster‐specific (Fig. 3a; Table S9). Among the few DEGs restricted to individual cell clusters, we identified *SECRETORY CARRIER MEMBRANE PROTEIN 5* (*SCAMP5*), a gene for a membrane trafficking protein that interacts with the TPLATE complex to regulate clathrin‐mediated endocytosis and that modulates the localization of aquaporins such as PLASMA INTRINSIC PROTEIN 2;1 (PIP2;1), thereby impacting stress responses including drought tolerance (Yperman *et al*., 2021). The role of SCAMP5 in vesicle trafficking suggests that it may regulate the turnover or localization of defense‐related proteins during pathogen infection. Another highly specific DEG was the gene for PLASMA INTRINSIC PROTEIN 1;4 (PIP1;4), an aquaporin that facilitates apoplastic H_2_O_2_ transport during defense against *Pseudomonas* (Tian *et al*., 2016). Both *SCAMP5* and *PIP1;4* were specifically activated in guard cells at 5 hpi (Fig. 3b), potentially reflecting a coordinated regulation of membrane trafficking and reactive oxygen species (ROS) signaling critical for stomatal defense during early infection stages.

Across mesophyll clusters, *Pst* DC3000 (EV)‐infected cells had substantially more DEGs than *Pst* DC3000 (AvrRpt2) cells at 3 hpi, with the numbers of DEGs at 5 hpi becoming more similar (Figs 2a, 3a; Table S10). There is, however, important nuance to these apparent differences: Volcano plots showed that 3 hpi DEGs in *Pst* DC3000 (EV) to have greater changes but lower significance, suggesting not only stronger but also more heterogeneous activation across individual cells in *Pst* DC3000 (EV) samples and weaker but more consistent changes in *Pst* DC3000 (AvrRpt2) cells (Fig. S6). This was different from the bulk protoplast RNA‐seq data at 4 hpi, in which the *Pst* DC3000 (AvrRpt2) samples had far more DEGs (Table S3). We interpret this difference as likely being due to averaging over many more cells in the bulk samples compared to the scRNA‐seq samples, providing more statistical power in the bulk samples to detect the typically smaller expression changes in ETI DEGs.

To understand the broader functional shifts, we performed *k*‐means clustering on all DEGs, defining gene modules with distinct expression profiles (Figs 3c, S7; Table S11). Module A2‐1, a JA/oxoacid/lipid‐metabolism gene set, was progressively repressed between 3 and 5 hpi. Concurrently, the photosynthesis/chloroplast‐related Module A2‐2 was unaffected at 3 hpi but declined by 5 hpi in nearly every cell cluster. By contrast, two defense‐related modules, Module A2‐3 and Module A2‐6, showed robust upregulation across all cell clusters at 3 hpi, with further amplification at 5 hpi, highlighting a broad but intensifying wave of defense (Fig. 3c).

Building on these module‐level dynamics, we identified the cell clusters with the earliest and strongest responses. The integrated Immunity Score, calculated from overall DEG patterns (Fig. 1d), pointed to Mesophyll_9 and Mesophyll_8 as the clusters that likely respond the earliest to *Pst* DC3000 (AvrRpt2). Further subdivision of these two populations into seven subclusters revealed specific patterns: The PRIMER cell marker gene *BON3* showed sparse expression in Mesophyll_8 and Mesophyll_9 at 3 and 5 hpi, which may be explained by late induction of *BON3* in PRIMER cells (Nobori *et al*., 2025). *ICS1*, a hallmark of SA biosynthesis, was upregulated at 3 hpi, with expression further concentrating in Mesophyll_8 at 5 hpi (Fig. 3d). Additionally, several key components of systemic acquired resistance (SAR), such as *ALD1*, *SAR DEFICIENT 4* (*SARD4*), and *FMO1*, were induced in the Mesophyll_8 cluster at 5 hpi (Fig. 3d). This localized induction is compatible with Mesophyll_8 acting similarly as a ‘bystander’ cell population, actively sensing and amplifying defense signals likely to be initiated by adjacent PRIMER cells.

We again used MINI‐EX (Ferrari *et al*., 2022) to identify top‐ranked regulons in each cell cluster (Figs 3e, S8; Tables S6, S12, S13). Even under pathogen exposure, the key cell identity network, controlled by TF FAMA/AT3G24140 (Ohashi‐Ito & Bergmann, 2006), remained among the most highly ranked regulons in guard cells.

Focusing on the central defense phytohormone SA, we examined TFs that regulate *ICS1* and *AVRPPHB SUSCEPTIBLE 3* (*PBS3*), the two key enzymes of SA biosynthesis. The known SA activators *SAR DEFICIENT 1* (*SARD1*) and *WRKY46* (Zhang *et al*., 2010; Wang *et al*., 2011) were specifically enriched in Mesophyll_10 together with the SA‐JA signaling hub TF gene *WRKY70* (Li *et al*., 2004), in *Pst* DC3000 (AvrRpt2) 5 hpi samples (Figs 3e, S8). We also noticed that *ICS1* expression was induced in Mesophyll_10 at 3 hpi and further enhanced at 5 hpi (Fig. 1c; Table S9).

At the same time, the SA‐responsive TF gene *WRKY75*, which promotes SA by antagonizing JA and promoting ROS (Li *et al*., 2004; Guo *et al*., 2017), was absent from Mesophyll_9 in the mock‐treated cells but was dramatically upregulated to define one of the top regulons at both 3 and 5 hpi (Fig. 3e). Coupled with the presence of a regulon regulated by GT‐3A/AT5G01380 (Fig. S8), a trihelix TF gene previously reported to govern defense in PRIMER cells (Nobori *et al*., 2025), this confirms Mesophyll_9 as a primary, early AvrRpt2‐responsive population. Conversely, the negative regulators *WRKY11* and *WRKY17*, which antagonize defense via JA‐dependent pathways (Journot‐Catalino *et al*., 2006), first appeared in Mesophyll_9 and later also in other clusters during *Pst* DC3000 (AvrRpt2) infection. The function of negative regulation of SA circuits during early defense remains, however, unclear (Fig. 3f).

WRKY70 also appeared among the top regulons in Mesophyll_11 and _12, and in epidermal and vascular clusters at both 3 and 5 hpi. A CBP60g‐related regulon was enriched in the epidermis (Fig. 3e), consistent with another recent study (Chhillar *et al*., 2025). Together, this suggested that the circuits underlying SA responsiveness and SA biosynthesis are activated in overlapping, but not identical cell populations: In Mesophyll_10, both SA response regulator *WRKY70* and the master regulators of SA biosynthesis *SARD1* and *WRKY46* are activated. By contrast, the SA response regulator *WRKY75* is only activated in Mesophyll_9, and the SA biosynthesis regulator *CBP60g* is only activated in the epidermis. Collectively, this set of TFs and the genes they regulate define complex cell‐cluster‐specific networks that modulate SA biosynthesis, SA response, and SA‐JA cross talk (Fig. 3f).

Together, these findings reveal a two‐tiered regulatory landscape during the very early onset of plant immunity: Core lineage regulons robustly preserve cell‐type identity under stress, while concurrently, shared defense and metabolic gene modules are deployed broadly but tuned to different extents in the timing and magnitude of change by each cell cluster.

### 4. The expression patterns of RLK, RLCK, and NLR genes are tuned by cell clusters

To determine in detail whether ETI and PTI components were differently deployed across cell clusters, we examined the expression patterns of key receptors in immunity: RLKs, RLCKs, which also include developmental regulators, and NLRs, which rarely seem to function in processes other than defense. From the known receptor repertoire (Shiu *et al*., 2004), we identified among the expressed genes in our data set a total of 350 RLK and 116 RLCK genes after excluding genes with low expression variance (see Section II), as well as 132 genes encoding NLR proteins (Tables S14–S16). We defined modules for RLKs, RLCKs, and NLRs, as we had done before for all genes.

**Fig. 4:**
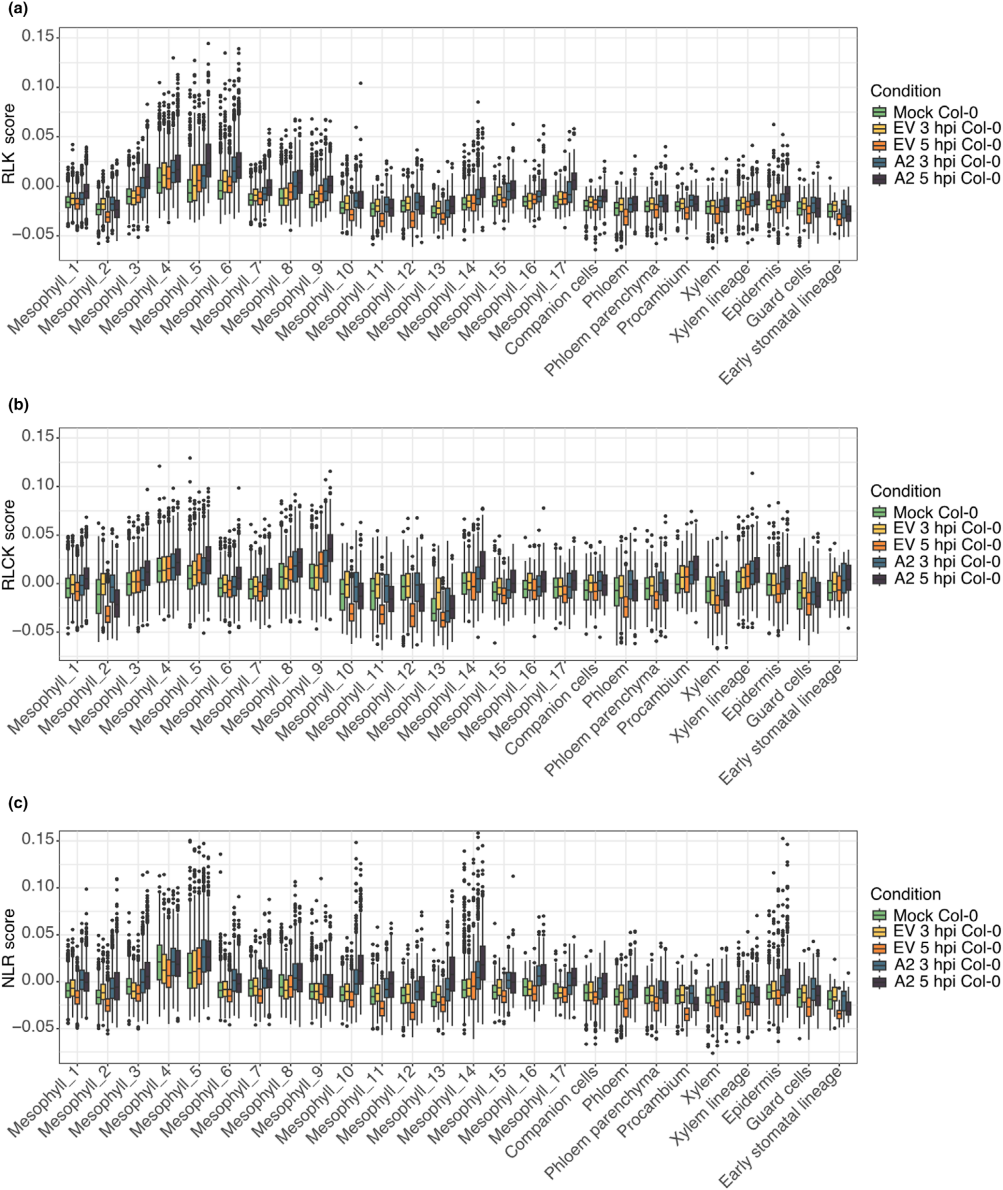
Cluster‐resolved expression of receptor‐like kinases (RLKs), receptor‐like cytoplasmic kinases (RLCKs), and nucleotide‐binding domain and leucine‐rich repeats (NLRs). Box line plots showing, for each cell cluster, the average module score of all RLK (a), RLCK (b), and NLR genes (c) in mock (green), EV 3 h post infection (hpi, yellow), EV 5 hpi (orange), A2 3 hpi (light teal), and A2 5 hpi (dark teal) samples. Center lines indicate medians; boxes span the IQR; whiskers extend to 1.5 × IQR. EV and A2 are short for *Pseudomonas syringae* pv Tomato (*Pst*) DC3000 (EV) and *Pst* DC3000 (AvrRpt2), respectively.

The average expression of RLK and RLCK genes increased at 3 hpi in both *Pst* DC3000 (EV) and *Pst* DC3000 (AvrRpt2) samples, but this induction was transient in *Pst* DC3000 (EV), returning to or even dropping below mock levels by 5 hpi across nearly all cell types except Mesophyll_3, _4, _5, _8, and _9. By contrast, *Pst* DC3000 (AvrRpt2) triggered a sustained and broader activation, with RLKs and RLCKs upregulated at 5 hpi in most cell types, except Mesophyll_2, _11, and _13, and guard cells. At the module level, RLK‐M1, RLK‐M3, and RLCK‐M1 genes were elevated at 3 hpi but decreased to mock levels by 5 hpi in *Pst* DC3000 (EV). By comparison, RLK‐M3 and RLCK‐M2 modules showed continued activation in 3 and 5 hpi in *Pst* DC3000 (AvrRpt2), while RLK‐M1 was similarly induced early in both treatments. RLK‐M2 genes were mainly upregulated in vascular cells at 5 hpi in both treatments (Figs 4a,b, S9a,b; Tables S14, S15, S17, S18).

Expression of NLR genes generally remained stable or slightly increased at 3 hpi and was reduced at 5 hpi in *Pst* DC3000 (EV), except for decreased expression in Mesophyll_3, _4, and _8 at 3 hpi and modest induction in Mesophyll_5 at 5 hpi. In *Pst* DC3000 (AvrRpt2), NLR genes, particularly those in NLR‐M4, were upregulated in a time‐dependent manner across multiple cell clusters (Figs 4c, S9c; Tables S16, S19).

Mesophyll_4 and _5 had higher basal expression levels of RLK, RLCK, and NLR genes already before pathogen challenge, as demonstrated by RLK‐M1, RLCK‐M1, and NLR‐M3 (Fig. S9; Tables S14–S16). Constitutively high expression of immune receptor and defense genes can impose fitness costs, often manifesting as growth retardation or reduced reproductive success (Huot *et al*., 2014; Karasov *et al*., 2017). The uneven expression patterns of RLK, RLCK, and NLR genes may reflect a mechanism that minimizes damage to cell types that are either less important for defense or that are particularly sensitive to activation of defense.

### 5. Temporal orchestration of immune and growth programs in distinct Arabidopsis leaf cell clusters

Returning to all genes, we had found that both *Pst* DC3000 (EV) and *Pst* DC3000 (AvrRpt2) infections induced similar gene modules, including photosynthesis‐related modules (Module EV‐2 and Module A2‐2) and defense‐related modules (Module EV‐4 and Module A2‐3). Cell‐type‐specific expression patterns of all these modules were already apparent in mock‐treated cells. To systematically characterize the relationship between photosynthesis‐ and defense‐related modules, we defined two core gene sets: a core growth module from the overlap of the photosynthesis‐related Module EV‐2 and Module A2‐2, and a core defense module from the overlap of Module EV‐4 and Module A2‐3.

**Fig. 5:**
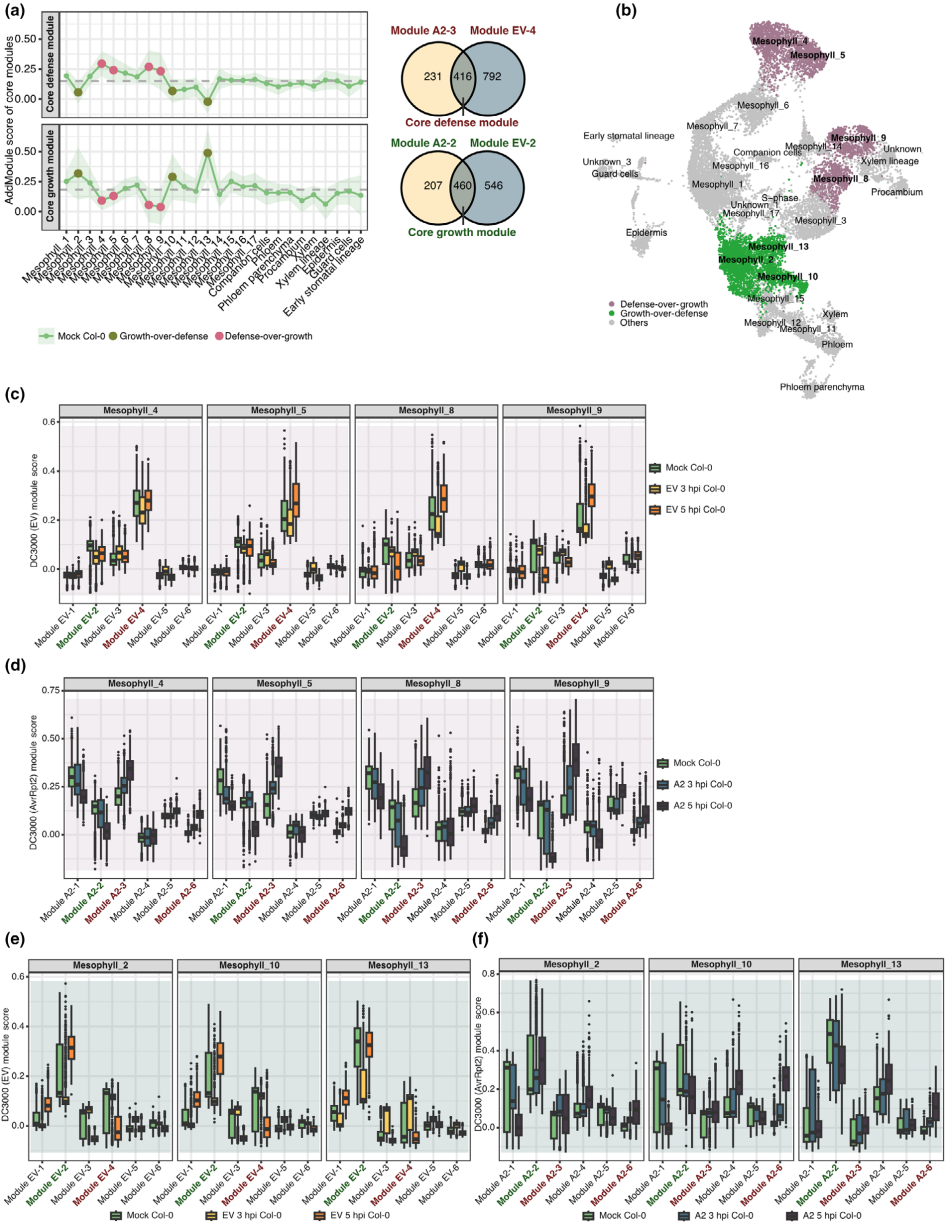
Distinct temporal trajectories during pattern‐triggered immunity (PTI) and effector‐triggered immunity (ETI). (a) Right: definition of a core defense module from the overlap of Modules A2‐3 and EV‐4, and of a core growth module from the overlap of Modules A2‐2 and EV‐2. Left: line plots showing the per‐cluster average scores for the core defense (upper) and core growth (lower) modules in mock (green) samples. Large colored circles highlight cell clusters with a ‘defense‐over‐growth’ (magenta) or ‘growth‐over‐defense’ (olive) phenotype. Shaded ribbons indicate mean ± SE. (b) Uniform manifold approximation and projection (UMAP) showing spatial distribution of ‘growth‐over‐defense’ clusters (green, Mesophyll_2, _10, _13) vs ‘defense‐over‐growth’ clusters (magenta, Mesophyll_4, _5, _8, _9) in the mock samples; all other clusters are in gray. (c–f). Boxplots of per‐cell module scores in the ‘defense‐over‐growth’ (c, d) and ‘growth‐over‐defense’ (e, f) cell clusters in (c, e) mock (dark green), EV 3 h post infection (hpi, gold), EV 5 hpi (orange) and (d, f) mock (dark green), A2 3 hpi (light teal) and A2 5 hpi (dark teal) samples. Within each panel, the growth‐related Modules EV‐2 and A2‐2 are labeled in green, and the defense‐related Modules EV‐4 and A2‐3, A2‐6 in red. Center lines indicate medians; boxes span the IQR; whiskers extend to 1.5 × IQR. EV and A2 are short for *Pseudomonas syringae* pv tomato (*Pst*) DC3000 (EV) and *Pst* DC3000 (AvrRpt2), respectively.

Mapping the two core modules onto the different cell clusters revealed three distinct mesophyll populations: ‘growth‐over‐defense’ clusters Mesophyll_2, _10, and _13 with high expression of the core growth module and low expression of the core defense module, and ‘defense‐over‐growth’ clusters Mesophyll_4, _5, _8, and _9, which showed the inverse pattern. A third group of clusters had an intermediate profile (Figs 5a,b, S10). Consistent with this observation, the phytosynthesis‐related genes *LHCB6*, *LHCB1.2*, and *LHCB1.3* were expressed more strongly in the ‘growth‐over‐defense’ clusters compared with other clusters (Fig. S11). Taken these findings together, we suggest that mesophyll cells can be specialized in prioritizing either photosynthetic efficiency or immune response.

In ‘defense‐over‐growth’ cell clusters, defense‐related modules were upregulated at 5 hpi upon both *Pst* DC3000 (EV) and *Pst* DC3000 (AvrRpt2) infection (Fig. 5c,d). By contrast, ‘growth‐over‐defense’ cell clusters showed different behaviors upon *Pst* DC3000 (EV) and *Pst* DC3000 (AvrRpt2) infections (Fig. 5e,f). Upon *Pst* DC3000 (EV) infection, these cells only transiently suppressed the growth‐related Module EV‐2 at 3 hpi (Fig. 5e), and recovered or even upregulated its activity by 5 hpi. The defense‐related Module EV‐4 was not induced except for modest activation in Mesophyll_13 at 3 hpi and had declined again by 5 hpi (Fig. 5e).

Upon *Pst* DC3000 (AvrRpt2) infection, most cells in both the ‘growth‐over‐defense’ and ‘defense‐over‐growth’ clusters activated the defense Modules A2‐3 and A2‐6 at both 3 and 5 hpi, and both types of clusters continued to repress the growth‐related Module A2‐2 also at 5 hpi (Fig. 5d,f), reflecting a robust ETI‐driven shift in gene activity.

These divergent transcriptomic trajectories highlight the intrinsic heterogeneity of leaf cells with either progrowth or prodefense features. We speculate that this could potentially contribute to early resistance or early susceptibility to infection with an avirulent pathogen.

### 6. Potential for alternative defense strategies in the *cue1*‐6 mutant

**Fig. 6:**
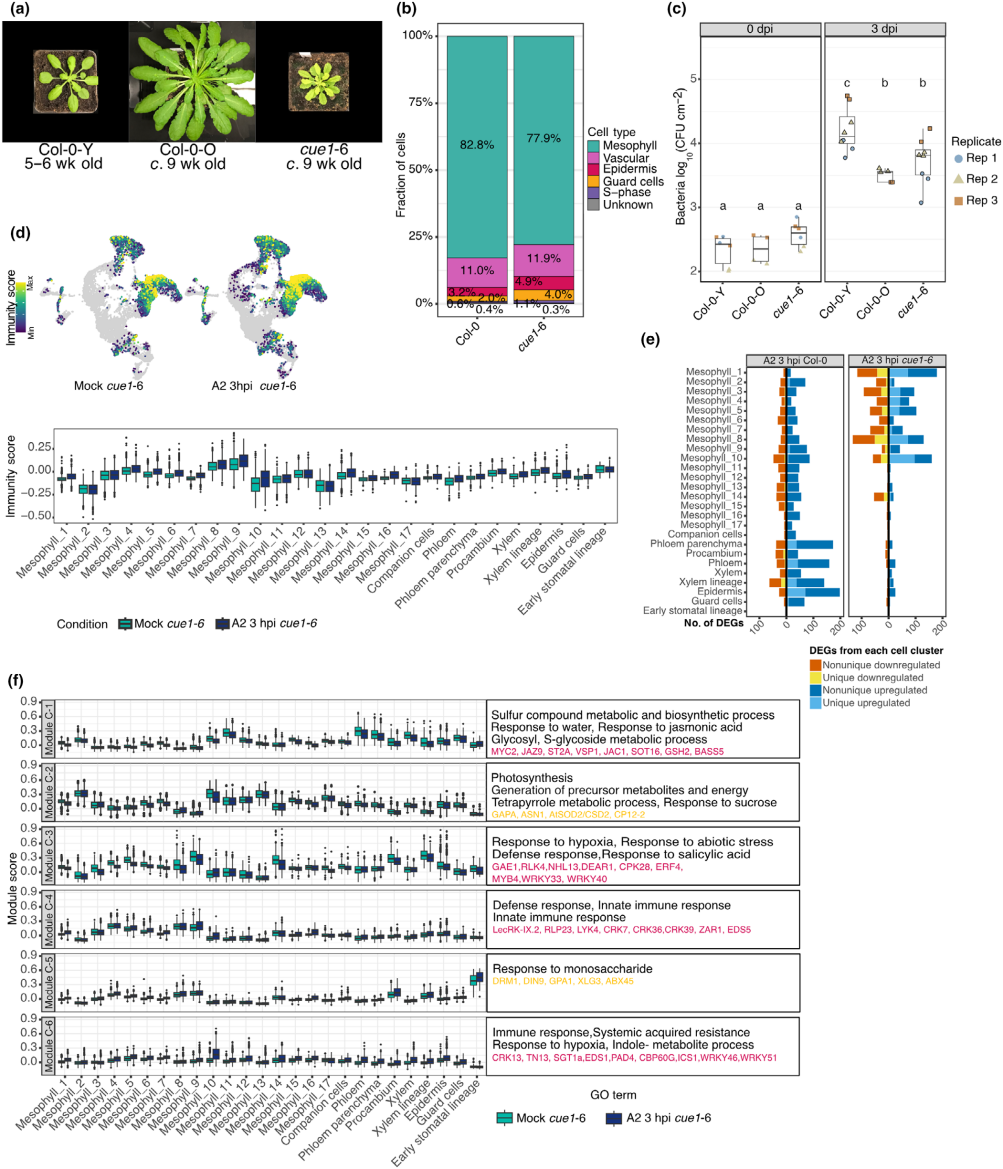
Early effector‐triggered immunity (ETI) responses in *cue1*‐6 mutants. (a) Rosette morphology of 5‐ to 6‐wk‐old Col‐0 (Col‐0‐Y), 9‐wk‐old Col‐0 (Col‐0‐O), and 9‐wk‐old *cue1*‐6 plants. (b) Bar chart of cell‐type proportions in Col‐0 and *cue1*‐6 samples. Colors denote major cell types: mesophyll (green), vasculature (magenta), epidermis (dark red), guard‐cell lineage (orange), S‐phase (purple), and unassigned/unknown (gray). (c) Bacterial growth assay. Leaves were infiltrated with *Pst* DC3000 (AvrRpt2) at OD_600_ = 0.0001, and bacterial colonization was quantified at 0 or 3 d post infection (dpi). Each dot represents one individual plant (2 or 3 leaves for Col‐0 and 6–8 leaves for *cue1*‐6). Colors and shapes represent independent experiments. Log CFU values were compared across sample groups. Different letters indicate significant differences (One‐way ANOVA, Tukey's HSD, *P* < 0.05). Center lines indicate medians; boxes span the IQR; whiskers extend to 1.5 x IQR. (d) Immunity Scores highlight the relative expression level of immune‐related differentially expressed genes (DEGs) (Fig. 1d) in each cell (upper, uniform manifold approximation and projection (UMAP)), or in each cell cluster (lower, boxplot). Center lines indicate medians; boxes span the IQR; whiskers extend to 1.5 × IQR. (e) Number of DEGs (*P*‐adj < 0.05, |log_2_FC| > 0.75) from each cell cluster of *cue1*‐6 plants infected with *Pst* DC3000 (AvrRpt2) at 3 h post infection (hpi) compared with the corresponding cell cluster in mock samples. The left chart shows the number of DEGs in Col‐0 after *Pst* DC3000 (AvrRpt2) infiltration at 3 hpi. Bars are stacked by DEG category, as indicated on the right. (f) Boxplots of average module score values for Modules C1–C6 from DEGs in (e) in *cue1*‐6 mock (green) and *cue1*‐6 A2 3 hpi (blue) samples. Gene ontology (GO) terms and representative top genes (highlighted in red, sucrose‐responsive genes are highlighted in yellow) for each module are listed alongside the panels. Center lines indicate medians; boxes span the IQR; whiskers extend to 1.5 × IQR. A2 is short for *Pseudomonas syringae* pv Tomato (*Pst*) DC3000 (AvrRpt2). CFU, colony‐forming units.

To investigate how a shift in leaf cell populations affects immunity, we made use of the *cue1*‐6 mutant, a reticulate mutant that has fewer mesophyll cells and a stunted growth phenotype (Fig 6a) (Streatfield *et al*., 1999; Staehr *et al*., 2014). In our scRNA‐seq data, *cue1*‐6 showed a reduced proportion of mesophyll cells compared to Col‐0 (not statistically significant; adj *P* = 0.2638) and a significantly increased proportion of guard cells (adj *P* = 0.0364) (Figs 6b, S12).

Because of their slower growth, we infected *cue1*‐6 mutants only after 9 wk of growth (Fig. 6a). Bacterial growth in *cue1*‐6 was comparable to that in older, age‐matched Col‐0 old plants (Col‐0‐O), but significantly lower than in younger, 5‐ to 6‐wk‐old Col‐0 young plants (Col‐0‐Y) (Fig. 6c), indicating that *cue1*‐6 has enhanced resistance relative to WT plants of similar size.

The Immunity Score was elevated across nearly all cell clusters in *cue1*‐6 infected with *Pst* DC3000 (AvrRpt2) (Fig. 6d). Notably, the number of DEGs unique to specific mesophyll clusters was higher than in Col‐0 (Fig. 6e; Table S20), suggesting that *cue1*‐6 has mechanisms that partially compensate for the altered mesophyll architecture. We categorized DEGs from all cell clusters into six co‐expression modules. These modules were similar to modules defined in Col‐0 infected with *Pst* DC3000 (AvrRpt2), with Module C‐1, enriched for JA response genes, and Module C‐2, associated with photosynthesis, being downregulated across most clusters; conversely, Modules C‐4 and C‐6, enriched for immune response genes, were upregulated in the majority of clusters (Figs 6f, S13; Table S21).

Modules C‐2 and C‐5 were enriched for genes involved in sucrose and monosaccharide responses, respectively (Fig. 6f), highlighting potentially an alternative defense strategy. Genes associated with sugar starvation and nitrogen remobilization, such as *GLUTAMINE‐DEPENDENT ASPARAGINE SYNTHASE 1* (*ASN1*), *DARK‐INDUCIBLE 9* (*DIN9*), and *ARABIDOPSIS THALIANA ACYL‐COA‐DIACYLGLYCEROL ACYLTRANSFERASE* (*ABX45*), were upregulated in Mesophyll_2, _5, and _1 respectively. By contrast, genes encoding regulators of the Calvin–Benson cycle, including *CP12 DOMAIN‐CONTAINING PROTEIN 2* (*CP12‐2*) and *GLYCERALDEHYDE 3‐PHOSPHATE DEHYDROGENASE A SUBUNIT* (*GAPA*) (Marri *et al*., 2005), displayed heterogeneous expression: *CP12‐2* was downregulated while *GAPA* was induced, suggesting local imbalances in photosynthetic carbon flux among different mesophyll cell populations. The chloroplastic redox enzyme *COPPER/ZINC SUPEROXIDE DISMUTASE 2* (*AtSOD2/CSD2*), which is crucial for ROS production and regulated by sucrose level (Dugas & Bartel, 2008), was consistently induced in several mesophyll clusters. Additionally, *G PROTEIN ALPHA SUBUNIT 1* (*GPA1*) and *EXTRA‐LARGE GTP‐BINDING PROTEIN 3* (*XLG3*), components of the heterotrimeric G‐protein complex that can respond to sucrose to regulate root growth (Huang *et al*., 2006; Ding *et al*., 2008), were upregulated in some mesophyll cell clusters (Fig. S14; Table S22). Given that *cue1*‐6 has reduced sucrose accumulation (Voll *et al*., 2003), these data suggest that modulation of sugar metabolism and signaling pathways may contribute to the early defense response against pathogens in this mutant.

We also examined the expression patterns of critical TF genes involved in SA and JA signaling across different cell clusters. Key SA‐related WRKY regulons, including those defined by WRKY75, WRKY17, WRKY11, WRKY70, and WRKY46, were detected in various *cue1*‐6 cell clusters, with expression patterns similar to those observed in Col‐0 WT. For example, the WRKY46 regulon showed specific induction in Mesophyll_10, while WRKY75 and WRKY17 regulons were predominantly activated in Mesophyll_9 after infection (Fig. S15; Tables S23, S24). Additionally, the PRIMER cell marker *GT‐3A* (Nobori *et al*., 2025)‐related regulon was enriched in Mesophyll_4, _8, and _9 in both mock and *Pst* DC3000 (AvrRpt2)‐infected samples, similar to its distribution in Col‐0 WT (Figs S8, S15).

Regarding JA signaling, the regulon controlled by the master TF MYC2, a well‐established activator of JA‐mediated defense responses, was among the top 10 regulons primarily induced in phloem tissues, but it was notably inactive in Mesophyll_14 and epidermal clusters (Fig. S15). Conversely, the regulon controlled by *JASMONATE ASSOCIATED MYC2 LIKE 2* (JAM2), a negative regulator of JA signaling that antagonizes MYC2 activity (Sasaki‐Sekimoto *et al*., 2013), was induced in the Mesophyll_4, _5, and _16 clusters. This complementary and spatially distinct expression patterns of MYC2 and JAM2 regulons suggest a complex, possibly noncell‐autonomous antagonistic regulation of JA signaling across cell types.

Together, these results demonstrate that despite developmental constraints, *cue1*‐6 mutants are resilient in their immune responses. Most core immune modules are similar to those in WT, but we also see a potentially alternative strategy: altered sucrose signaling. The cell cluster‐specific distribution of these TF regulons underscores the importance of cell‐type context in hormone‐mediated defense.

## IV. Discussion

A perennial topic in plant immunity is the balance between growth and defense. Clearly, different cell layers, such as epidermis and mesophyll, play very different roles in defense, and the growth‐defense trade‐off therefore is likely to play out differently in these layers (Wyrsch *et al*., 2015). In addition, there is division of labor among different mesophyll cells when it comes to processes such as photosynthesis (Xia *et al*., 2022). Here, we tested the hypothesis that growth‐defense trade‐offs are managed in a cell‐type‐specific manner.

### 1. Intrinsic mesophyll heterogeneity driving growth‐defense trade‐offs and shaping infection outcomes

The major cell type of leaves, mesophyll cells, features heterogeneous transcriptomic profiles influenced by the physical location of cells and their developmental state (Xia *et al*., 2022; Guo *et al*., 2025). Previous single‐cell transcriptome studies have already described cell‐type‐specific heterogeneity of pathogen responses in the Arabidopsis leaf (Delannoy *et al*., 2023; Tang *et al*., 2023; Zhu *et al*., 2023; Chhillar *et al*., 2025; Nobori *et al*., 2025). Our study, which specifically focused on mesophyll cells, identified resilient cell populations that quickly reinitiate programs supporting growth after pathogen attack.

We found that specific cell clusters with high expression of photosynthesis module genes consistently had low expression of defense module genes, and vice versa (Figs 5a, S10), indicating a cell‐intrinsic trade‐off. When defense scores are high during ETI, in *Pst* DC3000 (AvrRpt2) challenged cells, photosynthesis module scores are correspondingly reduced, reinforcing the notion of an antagonistic relationship (Fig. 5d). Conversely, growth‐over‐defense cells are delayed in the activation of defense (Fig. 5f). These observations suggest that individual cell populations, through cluster‐specific activation of defense‐associated transcription factors, calibrate their unique contributions to the overall immune response. In some cases, changes in gene expression are restricted to one cell type (lineage specificity); for example, *BGLU40* and *AT2G05540* are primarily upregulated in the epidermis (Fig. 2b), while in others, differences between cell types are more gradual. For example, a gene might be activated in two cell types, but with a delay in one of the cell types (temporal differences); the induction of *ICS1* first occurs in Mesophyll_9 and _8 and later in additional cell types (Fig. 1c), or activation might follow a similar temporal trajectory in two cell types, but being always lower in one of the cell types (amplitude differences), as seen for the different amplitude of activation of Module A2‐3 across cell types (Fig. 3c). We consider all three cases as examples of cell‐type‐specific gating of transcriptional responses.

Our data also illuminate how this trade‐off is expressed in the face of different challenges. Compared with the virulent *Pst* DC3000 (EV) strain, the avirulent *Pst* DC3000 (AvrRpt2) strain both suppresses photosynthetic activity and activates immunity more uniformly across mesophyll clusters, while the virulent *Pst* DC3000 (EV) strain leads to a more nuanced response. The expression of photosynthesis‐related genes is derepressed in growth‐over‐defense cells by 5 hpi, while defense gene expression is prioritized in defense‐over‐growth cells (Fig. 5). This observation suggests that virulent pathogen strategies apparently involve selective manipulation of host cellular priorities, an insight with potentially profound implications for engineering durable plant resistance that minimizes growth penalties.

The origins of this intrinsic heterogeneity, whether predominantly developmental, metabolic, or epigenetic, remain an open question worthy of future investigation. A priority should be the identification of TFs and the GRNs they regulate or other signaling factors that connect growth and defense.

### 2. Temporal coordination of immune modules determines resistance

Optimal resistance requires precise temporal and spatial orchestration of core defense modules. While Zhu *et al*. (2023) identified distinct immune and susceptible mesophyll states at later infection stages (16–48 hpi), our analysis reveals dynamic transcriptional reprogramming during the early phases of infection (3–5 hpi). *Pst* DC3000 (AvrRpt2) induces progressive amplification of defense signals, whereas the virulent *Pst* DC3000 (EV) strain is able to dampen initial responses at later time points (Figs 2, 3). Notably, photosynthesis genes are rapidly suppressed in resistant interactions, with the proviso that we do not know whether mesophyll cells recover metabolic activity once defense has subsided (Fig. 5).

We identified the Mesophyll_9 cluster as a putative PRIMER cell population, Mesophyll_8 as bystander cells (Nobori *et al*., 2025). Apparent PRIMER cells in our data set are characterized by expression of genes for the transcription factors GT‐3A, WRKY75, and WRKY11/17, well‐known SA regulators. The SA‐producing Mesophyll_10 cluster is enriched in another set of transcription factor genes, encoding CBP60g, SARD1, and WRKY46, while key components of SAR are induced in Mesophyll_8 (Fig. 3e,f). Future spatial transcriptomics experiments could elucidate how these populations are spatially arranged and thus how their physical proximity might contribute to immunity outcomes.

### 3. Induced and natural genetic variation as untapped resources for the study of plant immunity

Natural and induced genetic variants of plants have been essential for dissecting the mechanisms underlying plant immunity, yet their potential for understanding immunity at single‐cell resolution remains largely untapped. Bulk transcriptomic studies in Arabidopsis have demonstrated how phytohormone mutants can be used to reveal layers of immune responses: the *dde2 ein2 pad4 sid2* quadruple mutant, impaired in JA/ethylene/PAD4/SA signaling, has informed how we think about gene regulation during PTI and ETI (Mine *et al*., 2018). Analyses of helper NLR mutants have revealed redundant as well as unequal contributions of these factors to PTI‐ and ETI‐ induced transcriptional changes (Saile *et al*., 2020).

Genetics has already been used on the pathogen side for single‐cell analyses, for example in a recent study that contrasted virulent and avirulent pathogens and that also compared two distinct effectors, AvrRpt2 and AvrRpm1 (Nobori *et al*., 2025). This study, as well as our study, has revealed genes that appear to control the behavior of different cell types and clusters, and scRNA‐seq analysis of plants mutant for these regulators will likely be very informative. In addition, while bulk RNA‐seq experiments have shown broadly similar patterns of early transcriptional reprogramming induced by different PAMPs or bacterial strains (Bjornson *et al*., 2021; Maier *et al*., 2021; Keppler *et al*., 2025), only single‐cell approaches can reveal how individual cell types decode these signals. Another avenue will be the comparison of CNL‐ and TNL‐triggered defenses.

We have studied here the *cue1* mutant, which has altered leaf architecture and which is impaired in the shikimate pathway (Li *et al*., 1995; Voll *et al*., 2003). We found *cue1* to mount a robust ETI across all mesophyll clusters, but also, unexpectedly, to engage a sucrose module in the early hours of infection (Fig. 6d,f). Upregulation of *ASN1*, *DIN9*, and *AtSOD2* indicates activation of a sugar‐deprivation and ROS‐responsive network, while induction of *GPA1* and *XLG3* links this metabolic state to defense signaling cascades. These results suggest that sugar availability acts as an upstream cue modulating early immune activation, providing a mechanistic basis for the observed cross talk between primary metabolism and immunity (Fig. S14). Consistent with recent evidence that sugar activates defense‐related genes and sugar influx contributes to PTI (Yamada & Mine, 2024), these results highlight that developmental status and metabolic context can profoundly shape the immune landscape within specific mesophyll populations during early ETI events, potentially activating alternative defense strategies based on the plant's physiological condition. This bodes well for the use of natural genetic variation to better understand plant immunity at the single‐cell level, given what is already known from bulk RNA‐seq experiments (Corwin *et al*., 2016).

Looking forward, an important question will be how robust core PTI and ETI GRNs are in different mutant backgrounds or different environments. By systematically profiling mutants and natural accessions with altered environmental responses, growth, metabolism, or development, we can map how these processes are integrated with plant immunity, potentially uncovering previously underappreciated points of cross talk. We believe that single‐cell driven knowledge will enhance the field's ability to engineer crops with a better balance between optimal yield and pathogen resistance.

## Competing interests

DW holds equity in Computomics, which advises plant breeders. DW has also consulted for KWS SE, a globally active plant breeder and seed producer. The other authors declare no competing interests.

## Author contributions

SW and DW planned and designed the research. SW and HG performed experiments. SW, IB and P‐JW conducted data analysis. SW and DW wrote the manuscript. IB, P‐JW, HG, and MT reviewed and edited the manuscript and provided discussions.

## Disclaimer

The New Phytologist Foundation remains neutral with regard to jurisdictional claims in maps and in any institutional affiliations.

## Supporting information


**Fig. S1** Overview of the scRNA‐seq experimental design.
**Fig. S2** Single‐cell atlas of individual samples and marker genes for cell‐type annotation.
**Fig. S3** Single‐cell distribution of defense‐response signatures.
**Fig. S4** Gene modules in *Pst* DC3000 (EV) infected Col‐0 samples.
**Fig. S5** Distribution of the top 10 regulons across cell clusters in *Pst* DC3000 (EV) infected Col‐0 samples.
**Fig. S6** Distribution of DEGs across cell types in *Pst* DC3000 (EV) and *Pst* DC3000 (AvrRpt2) samples.
**Fig. S7** Gene modules in *Pst* DC3000 (AvrRpt2) infected Col‐0 samples.
**Fig. S8** Distribution of the top 10 regulons across cell clusters in *Pst* DC3000 (AvrRpt2) infected Col‐0 samples.
**Fig. S9** Expression patterns of RLKs, RLCKs and NLRs in *Pst* DC3000 (EV) and *Pst* DC3000 (AvrRpt2) infected Col‐0 samples.
**Fig. S10** Expression of core defense and growth module genes in mock and *Pst* DC3000 (EV) or *Pst* DC3000 (AvrRpt2) infected Col‐0 and *cue1*‐6 samples.
**Fig. S11** Expression pattern of photosynthesis‐related genes.
**Fig. S12** Cell‐type proportions in Col‐0 vs *cue1*‐6.
**Fig. S13** Gene modules in *Pst* DC3000 (AvrRpt2) infected 3 hpi *cue1*‐6 samples.
**Fig. S14** Sugar‐related DEGs from *Pst* DC3000 (AvrRpt2) infected samples at 3 hpi in *cue1*‐6.
**Fig. S15** Distribution of the top 10 regulons across cell clusters in *Pst* DC3000 (AvrRpt2) infected *cue1*‐6 samples.

**Table S1** Seurat clusters and corresponding cell type assignments.
**Table S2** Known marker genes for assignments of *Arabidopsis thaliana* leaf cell types.
**Table S3** Differentially expressed gene (DEG) list from bulk RNA‐seq of protoplasts from Arabidopsis leaves infected with *Pst* DC3000 (EV) or *Pst* DC3000 (AvrRpt2).
**Table S4** DEGs from each cell cluster in Pst DC3000 (EV) 3 hpi/Col‐0 vs mock/Col‐0 and 5 hpi/Col‐0 vs mock/Col‐0.
**Table S5** Gene list for Module EV‐1 to 6. DEGs across cell clusters: *Pst* DC3000 (EV) 3 hpi/Col‐0 vs mock/Col‐0 and 5 hpi/Col‐0 vs mock/Col‐0 were grouped into 6 modules.
**Tables S6–S8** Ranked Regulons predicted by MINI‐EX for each cell cluster in Mock Col‐0 (Table S6), *Pst* DC3000 (EV) 3 hpi Col‐0 (Table S7) and *Pst* DC3000 (EV) 5 hpi Col‐0 (Table S8) samples.
**Table S9** DEGs from each cell cluster in *Pst* DC3000 (AvrRpt2) 3 hpi/Col‐0 vs mock/Col‐0, 5 hpi/Col‐0 vs mock/Col‐0.
**Table S10** Number of DEGs from each cell cluster in *Pst* DC3000 (EV) and *Pst* DC3000 (AvrRpt2) at 3 and 5 hpi in Col‐0 samples.
**Table S11** Gene list for Module A2‐1 to 6. DEGs across clusters: *Pst* DC3000 (AvrRpt2) 3 hpi/Col‐0 vs mock/Col‐0 and 5 hpi/Col‐0 vs mock/Col‐0 were grouped into 6 modules.
**Tables S12, S13** Ranked Regulons predicted by MINI‐EX for each cell cluster in *Pst* DC3000 (AvrRpt2) 3 hpi Col‐0, *Pst* DC3000 (AvrRpt2) 5 hpi Col‐0 samples.
**Tables S14–S16** RLK (Table S14), RLCK (Table S15) and NLR (Table S16) genes examined in this study.
**Tables S17–S19** Pairwise statistical comparison of RLK (Table S17), RLCK (Table S18) and NLR (Table S19) module scores across treatments.
**Table S20** DEGs from each cell cluster in *Pst* DC3000 (AvrRpt2) 3 hpi/*cue1*‐6 vs mock/*cue1*‐6 mutant.
**Table S21** Gene list for Module C‐1 to 6. DEGs across cell clusters: *Pst* DC3000 (AvrRpt2) 3 hpi/*cue1*‐6 vs mock/*cue1*‐6 were grouped into 6 modules.
**Table S22** Sugar‐related DEGs from *Pst* DC3000 (AvrRpt2) 3 hpi *cue1*‐6 samples.
**Tables S23, S24** Ranked Regulons predicted by MINI‐EX for each cell cluster in Mock *cue1*‐6 (Table S23) and *Pst* DC3000 (AvrRpt2) 3 hpi *cue1*‐6 (Table S24) samples.
Please note: Wiley is not responsible for the content or functionality of any Supporting Information supplied by the authors. Any queries (other than missing material) should be directed to the *New Phytologist* Central Office.

## Data Availability

Sequencing reads have been deposited in the European Nucleotide Archive (ENA) (PRJEB94524 for scRNA‐seq, PRJEB94525 for bulk RNA‐seq). Scripts, code, and the scRNA‐seq processed dataset are available at doi: 10.5281/zenodo.16533111.
