# Supplementary material for: Cell‐type‐specific gating of gene regulatory modules as a hallmark of early immune responses in Arabidopsis leaves
Source: New Phytol. 2026 Jan 7;250(4):2007–25. doi: 10.1111/nph.70858 (PMC13103434; doi:10.1111/nph.70858)
Supplement: Supplementary file 1 — Fig. S1 Overview of the scRNA‐seq experimental design. Fig. S2 Single‐cell atlas of individual samples and marker genes for cell‐type annotation. Fig. S3 Single‐cell distribution of defense‐response signatures. Fig. S4 Gene modules in Pst DC3000 (EV) infected Col‐0 samples. Fig. S5 Distribution of the top 10 regulons across cell clusters in Pst DC3000 (EV) infected Col‐0 samples. Fig. S6 Distribution of DEGs across cell types in Pst DC3000 (EV) and Pst DC3000 (AvrRpt2) samples. Fig. S7 Gene modules in Pst DC3000 (AvrRpt2) infected Col‐0 samples. Fig. S8 Distribution of the top 10 regulons across cell clusters in Pst DC3000 (AvrRpt2) infected Col‐0 samples. Fig. S9 Expression patterns of RLKs, RLCKs and NLRs in Pst DC3000 (EV) and Pst DC3000 (AvrRpt2) infected Col‐0 samples. Fig. S10 Expression of core defense and growth module genes in mock and Pst DC3000 (EV) or Pst DC3000 (AvrRpt2) infected Col‐0 and cue1‐6 samples. Fig. S11 Expression pattern of photosynthesis‐related genes. Fig. S12 Cell‐type proportions in Col‐0 vs cue1‐6. Fig. S13 Gene modules in Pst DC3000 (AvrRpt2) infected 3 hpi cue1‐6 samples. Fig. S14 Sugar‐related DEGs from Pst DC3000 (AvrRpt2) infected samples at 3 hpi in cue1‐6. Fig. S15 Distribution of the top 10 regulons across cell clusters in Pst DC3000 (AvrRpt2) infected cue1‐6 samples. [file NPH-250-2007-s001.pdf]

New Phytologist **Supporting Information**

Article title: Cell-type specific gating of gene regulatory modules as a hallmark of early immune responses in Arabidopsis leaves

Authors: Shanshan Wang[1†], Ilja Bezrukov[1], Pin-Jou Wu[1,2], Hannah Gauß[1], Marja Timmermans[2], Detlef Weigel[1,3†]

Article acceptance date: 1 December 2025

The following Supporting Information is available for this article:

Supplementary Figures

Fig. S1 Overview of the scRNA-seq experimental design.

Fig. S2 Single-cell atlas of individual samples and marker genes for cell-type annotation.

Fig. S3 Gene modules in *Pst* DC3000 (EV) infected Col-0 samples.

Fig. S4 Gene modules in *Pst* DC3000 (EV) infected Col-0 samples.

Fig. S5 Distribution of the top 10 regulons across cell clusters in *Pst* DC3000 (EV) infected Col-0 samples.

Fig. S6 Distribution of DEGs across cell types in Pst DC3000 (EV) and *Pst* DC3000 (AvrRpt2) samples.

Fig. S7 Gene modules in *Pst* DC3000 (AvrRpt2) infected Col-0 samples.

Fig. S8 Distribution of the top 10 regulons across cell clusters in *Pst* DC3000 (AvrRpt2) infected Col-0 samples.

Fig. S9 Expression patterns of RLKs, RLCKs and NLRs in *Pst* DC3000 (EV) and *Pst* DC3000(AvrRpt2) infected Col-0 samples.

Fig. S10 Expression of core defense and growth module genes in mock and *Pst* DC3000 (EV) or *Pst* DC3000 (AvrRpt2) infected Col-0 and *cue1*-6 samples.

Fig. S11 Expression pattern of photosynthesis-related genes.

Fig. S12 Cell-type proportions in Col-0 versus *cue1*-6.

Fig. S13 Gene modules in *Pst* DC3000 (AvrRpt2) infected *cue1*-6 samples.

Fig. S14 Sugar-related DEGs from *Pst* DC3000 (AvrRpt2) infected samples at 3 hpi in *cue1*-6.

Fig. S15 Distribution of the top 10 regulons across cell clusters in *Pst* DC3000 (AvrRpt2) infected *cue1*-6 samples.

Supplementary Tables

Table S1 Seurat clusters and corresponding cell type assignments.

Table S2 Known marker genes for assignments of *Arabidopsis thaliana* leaf cell types.

Table S3 Differentially expressed gene (DEG) list from bulk RNA-seq of protoplasts from Arabidopsis leaves infected with *Pst* DC3000 (EV) or *Pst* DC3000 (AvrRpt2).

Table S3-1 Up-regulated and down-regulated gens in *Pst* DC3000 (AvrRpt2) vs Mock at 4 hpi from bulk protoplasts RNA seq data.

Table S3-2 Up-regulated and down-regulated genes in *Pst* DC3000 (EV) vs Mock at 4 hpi from bulk protoplasts RNA seq data. hpi, hours post infection.

Table S4 DEGs from each cell cluster in *Pst* DC3000 (EV) 3 hpi/Col-0 vs. mock/Col-0 and 5 hpi/Col-0 vs mock/Col-0. hpi, hours post infection.

Table S5 Gene list for Module EV-1 to 6. DEGs across cell clusters: *Pst* DC3000 (EV) 3 hpi/Col-0 vs. mock /Col-0 and 5 hpi/Col-0 vs. mock/Col-0 were grouped into 6 modules.

Table S6, S7, S8 Ranked Regulons predicted by MINI-EX for each cell cluster in Mock Col-0 (Table S6), *Pst* DC3000 (EV) 3hpi Col-0 (Table S7) and *Pst* DC3000 (EV) 5hpi Col-0 (Table S8) samples.

Table S9 DEGs from each cell cluster in *Pst* DC3000 (AvrRpt2) 3 hpi/Col-0 vs. mock/Col-0, 5 hpi/Col-0 vs mock/Col-0.

Table S10 Gene list for Module A2-1 to 6. DEGs across clusters: *Pst* DC3000 (AvrRpt2) 3 hpi/Col-0 vs. mock/Col-0 and 5 hpi/Col-0 vs. mock /Col-0 were grouped into 6 modules.

Table S11 Number of DEGs from each cell cluster in *Pst* DC3000 (EV) and *Pst* DC3000 (AvrRpt2) at 3 hpi and 5 hpi in Col-0 samples.

Table S12, S13 Ranked Regulons predicted by MINI-EX for each cell cluster in *Pst* DC3000 (AvrRpt2) 3hpi Col-0, *Pst* DC3000 (AvrRpt2) 5 hpi Col-0 samples.

Table S14, S15, S16 RLK (Table S14), RLCK (Table S15) and NLR (Table S16) genes examined in this study.

Relative expression levels of RLK (Table S14), RLCK (Table S15) and NLR (Table S16) genes grouped by their k-means modules in mock, *Pst* DC3000 (EV) 3 hpi/5 hpi, and *Pst* DC3000 (AvrRpt2) 3 hpi/5 hpi Col-0 samples. Data correspond to the heatmap shown in Fig. S9. RLK, receptor-like kinases; RLCK, receptor-like cytoplasmic kinases; NLR, nucleotide-binding domain and leucine-rich repeat; hpi, hours post infection.

Table S17, S18, S19 Pairwise statistical comparison of RLK (Table S17), RLCK (Table S18) and NLR (Table S19) module scores across treatments.

Pairwise statistical comparison of RLK (Table S17), RLCK (Table S18) and NLR (Table S19) module scores in mock, *Pst* DC3000 (EV) 3 hpi/5 hpi, and *Pst* DC3000 (AvrRpt2) 3 hpi/5 hpi Col-0 samples (related Fig. 4A). For each pairwise comparison, the table reports sample sizes (n_1,

n_2), test statistic, raw p-value, adjusted p-value (Benjamini–Hochberg correction), significance level, and group-wise mean and median RLK/RLCK/NLR relative expression level. Related to Table S17, S18, S19 respectively. RLK, receptor-like kinases; RLCK, receptor-like cytoplasmic kinases; NLR, nucleotide-binding domain and leucine-rich repeat; hpi, hours post infection.

Table S20 DEGs from each cell cluster in *Pst* DC3000 (AvrRpt2) 3 hpi/cue1-6 vs. mock/*cue1*-6 mutant.

Table S21 Gene list for Module C-1 to 6. DEGs across cell clusters: *Pst* DC3000 (AvrRpt2) 3 hpi/cue1-6 vs. mock /cue1-6 were grouped into 6 modules.

Table S22 Sugar-related DEGs from *Pst* DC3000 (AvrRpt2) 3 hpi *cue1*-6 samples.

Fold change and adjusted *p* value of sugar-related DEGs from *Pst* DC3000 (AvrRpt2) 3 hpi/cue1-6 vs. mock/cue1-6 (related to Fig.S14).

Table S23, S24 Ranked Regulons predicted by MINI-EX for each cell cluster in Mock cue1-6 (Table S23) and *Pst* DC3000 (AvrRpt2) 3 hpi cue1-6 (Table S24) samples.

Supplementary Figures

(a)

sample	time	genotype	number of cells	mean counts	mean genes	replicate
Mock		Col-0	20192	5882	1923	3
DC3000 (EV)	3 hpi	Col-0	10860	6462	2208	2
DC3000 (EV)	5 hpi	Col-0	15792	5170	1877	2
DC3000 (A2)	3 hpi	Col-0	14884	6637	2289	2
DC3000 (A2)	5 hpi	Col-0	15817	7332	2312	2
Mock		*cue1*-6	14480	7587	2181	2
DC3000 (A2)	3 hpi	*cue1*-6	17167	8421	2341	2

(b)

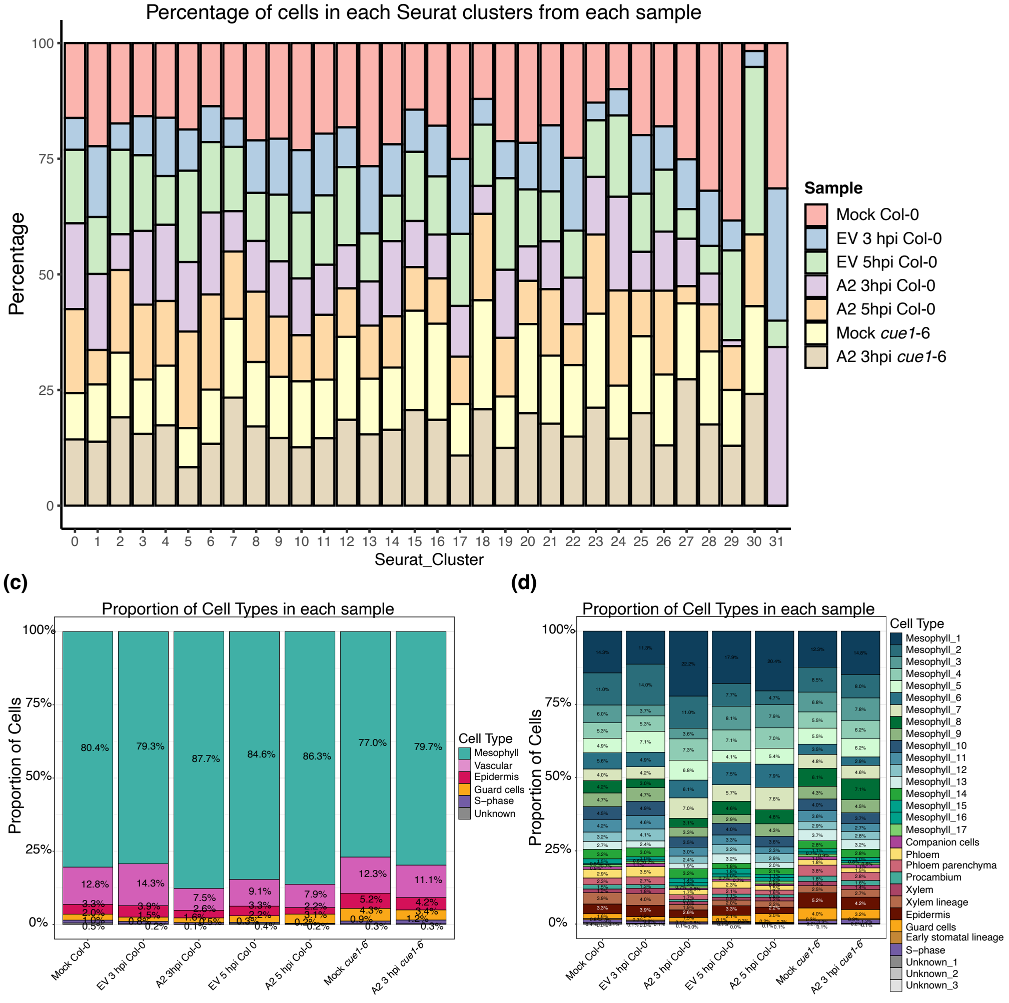

Fig. S1 | Overview of the scRNA-seq experimental design. (a) Summary of scRNA-seq data from samples at 3 and 5 hpi of mock, *Pst* DC3000 (EV) and *Pst* DC3000 (AvrRpt2) in Col-0 wild-type and *cue1*-6 mutant plants. Mean counts are mean counts of unique molecular identifiers (UMIs) per cell, mean genes are mean number of genes for which transcripts were detected per cell. (b) Cluster composition by sample. The proportion of cells from samples described in (a) in each cluster. (c) and (d) Proportion of cells in each cell type (labeled on the right) from samples

at 3 and 5 hpi of mock, *Pst* DC3000 (EV) and *Pst* DC3000 (AvrRpt2) in Col-0 wild-type and *cue1*-6 mutant plants. EV and A2 are short for *Pst* DC3000 (EV) and *Pst* DC3000 (AvrRpt2), respectively. Hpi, hours post infection.

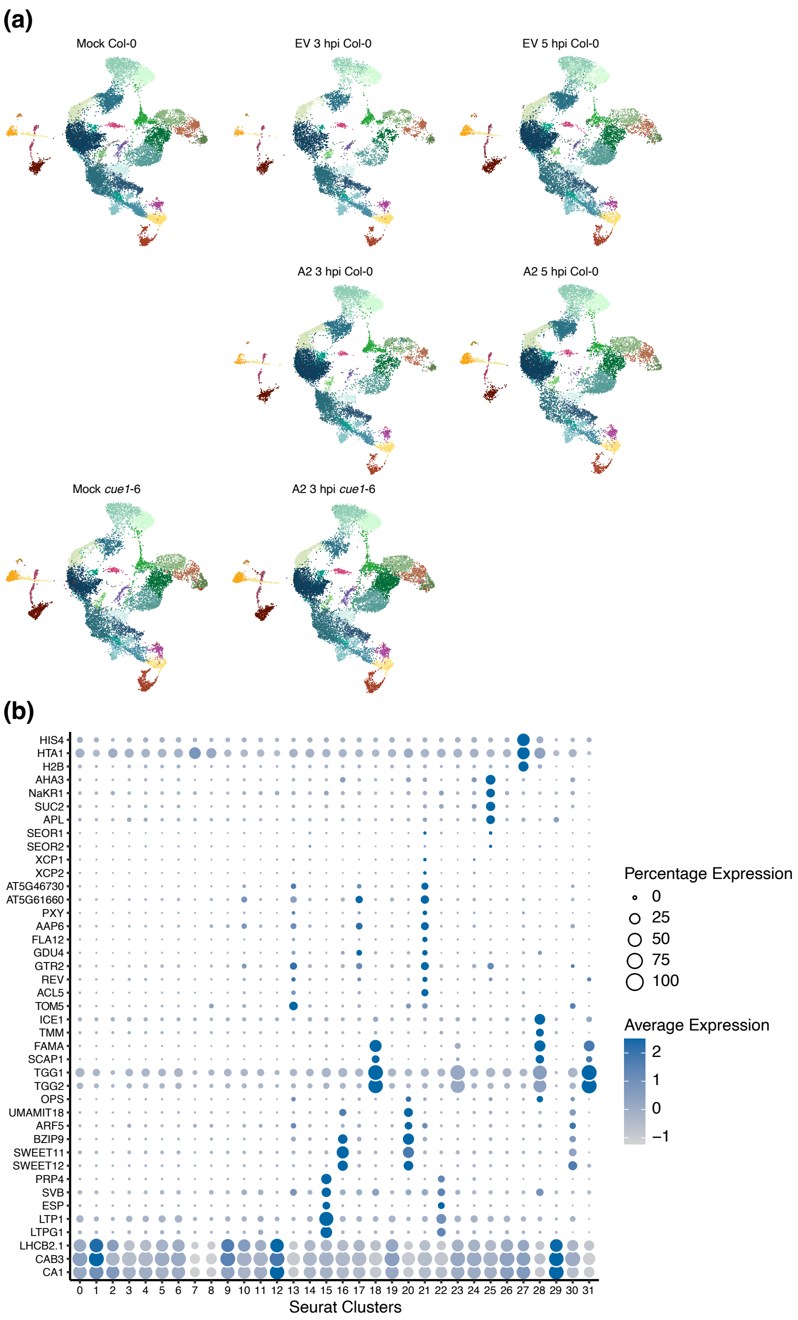

Fig. S2 | Single-cell atlas of individual samples and marker genes for cell-type annotation. (a) Uniform Manifold Approximation and Projection (UMAPs) for each sample, with biological replicates combined. EV and A2 are short for *Pst* DC3000 (EV) and *Pst* DC3000 (AvrRpt2), respectively. (b) Dot plot of known cell type marker genes across Seurat clusters: dot size indicates the fraction of cells expressing each gene, and colors represent scaled average expression values. Table S1 shows the marker genes and corresponding cell types. EV and A2

are short for *Pseudomonas syringae* pv. Tomato (*Pst*) DC3000 (EV) and *Pst* DC3000 (AvrRpt2), respectively. Hpi, hours post infection.

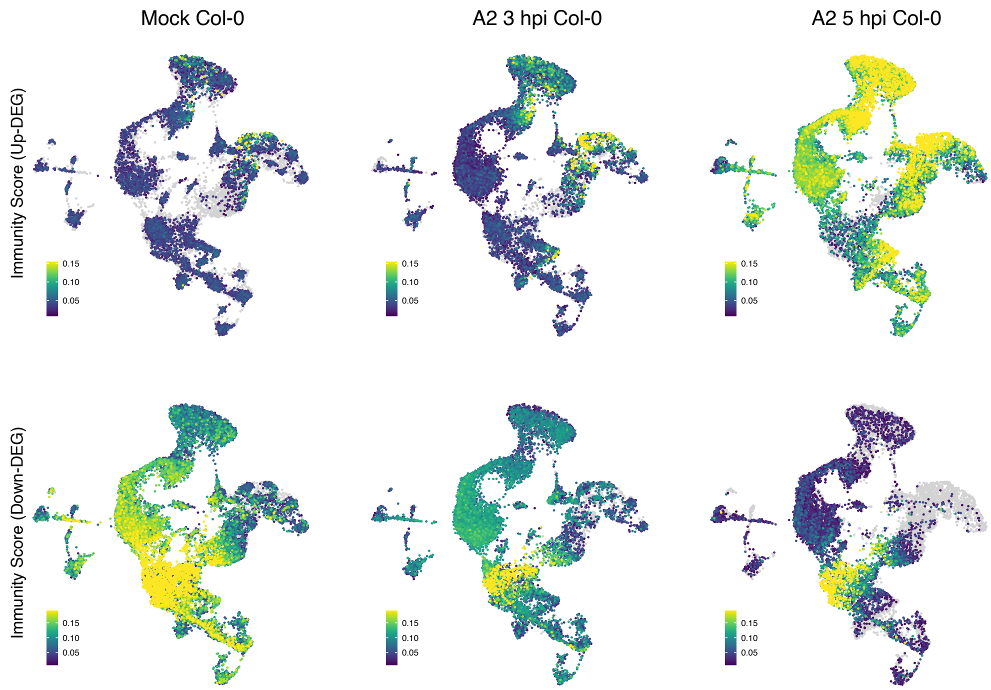

Fig. S3 | Single-cell distribution of defense-response signatures. UMAPs colored by an Immunity Score calculated for each cell as the mean scaled expression of immune-related DEGs identified by bulk-RNA-seq (see Fig. 1). Top row: score based on genes up-regulated by AvrRpt2 (*Up-DEG score*). Bottom row: score based on genes down-regulated by AvrRpt2 (*Down-DEG score*). Yellow denotes cells with higher average expression of the indicated gene set, and dark blue denotes lower expression. A2 is short for *Pseudomonas syringae* pv. Tomato (*Pst*) DC3000 (AvrRpt2). DEG, differentially expressed gene; UMAP, uniform manifold approximation and projection; hpi, hours post infection.

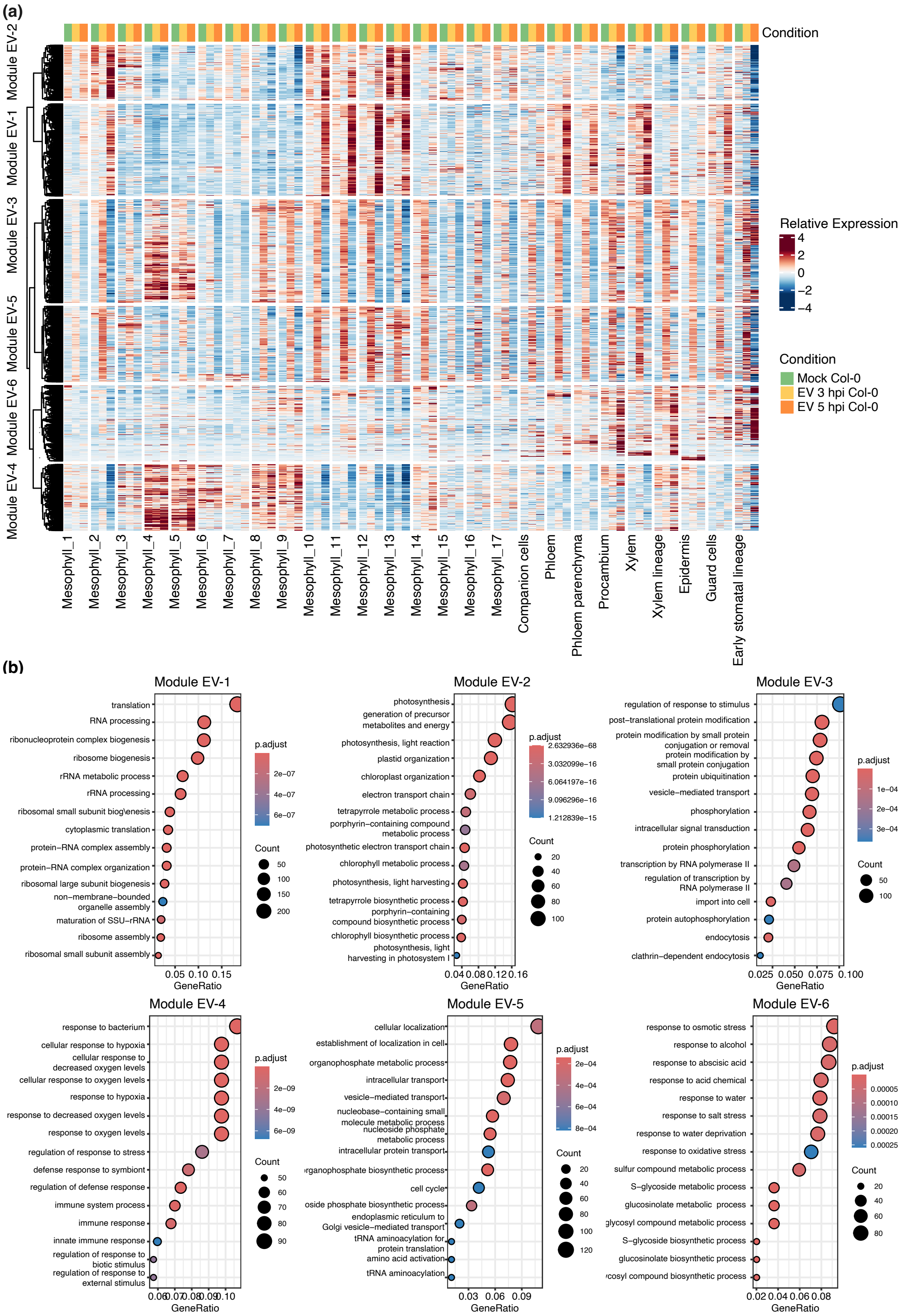
(a)
Condition
Module EV-2
Module EV-1
Module EV-3
Module EV-5
Module EV-6
Module EV-4
Relative Expression
4
2
0
−2
−4
Condition
Mock Col-0
EV 3 hpi Col-0
EV 5 hpi Col-0
Mesophyll_1
Mesophyll_2
Mesophyll_3
Mesophyll_4
Mesophyll_5
Mesophyll_6
Mesophyll_7
Mesophyll_8
Mesophyll_9
Mesophyll_10
Mesophyll_11
Mesophyll_12
Mesophyll_13
Mesophyll_14
Mesophyll_15
Mesophyll_16
Mesophyll_17
Companion cells
Phloem
Phloem parenchyma
Procambium
Xylem
Xylem lineage
Epidermis
Guard cells
Early stomatal lineage
(b)
Module EV-1
translation
RNA processing
ribonucleoprotein complex biogenesis
ribosome biogenesis
rRNA metabolic process
rRNA processing
ribosomal small subunit biog\enesis
cytoplasmic translation
protein−RNA complex assembly
protein−RNA complex organization
ribosomal large subunit biogenesis
non−membrane−bounded organelle assembly
maturation of SSU−rRNA
ribosome assembly
ribosomal small subunit assembly
GeneRatio
p.adjust
Count
Module EV-2
photosynthesis
generation of precursor metabolites and energy
photosynthesis, light reaction
plastid organization
chloroplast organization
electron transport chain
tetrapyrrole metabolic process
porphyrin−containing compound metabolic process
photosynthetic electron transport chain
chlorophyll metabolic process
photosynthesis, light harvesting
tetrapyrrole biosynthetic process
porphyrin−containing compound biosynthetic process
chlorophyll biosynthetic process
photosynthesis, light harvesting in photosystem I
Module EV-3
regulation of response to stimulus
post−translational protein modification
protein modification by small protein conjugation or removal
protein modification by small protein conjugation
protein ubiquitination
vesicle−mediated transport
phosphorylation
intracellular signal transduction
protein phosphorylation
transcription by RNA polymerase II
regulation of transcription by RNA polymerase II
import into cell
protein autophosphorylation
endocytosis
clathrin−dependent endocytosis
Module EV-4
response to bacterium
cellular response to hypoxia
cellular response to decreased oxygen levels
cellular response to oxygen levels
response to hypoxia
response to decreased oxygen levels
response to oxygen levels
regulation of response to stress
defense response to symbiont
regulation of defense response
immune system process
immune response
innate immune response
regulation of response to biotic stimulus
regulation of response to external stimulus
Module EV-5
cellular localization
establishment of localization in cell
organophosphate metabolic process
intracellular transport
vesicle−mediated transport
nucleobase−containing small molecule metabolic process
nucleoside phosphate metabolic process
intracellular protein transport
organophosphate biosynthetic process
cell cycle
nucleoside phosphate biosynthetic process
endoplasmic reticulum to Golgi vesicle−mediated transport
tRNA aminoacylation for protein translation
amino acid activation
tRNA aminoacylation
Module EV-6
response to osmotic stress
response to alcohol
response to abscisic acid
response to acid chemical
response to water
response to salt stress
response to water deprivation
response to oxidative stress
sulfur compound metabolic process
S−glycoside metabolic process
glucosinolate metabolic process
glycosyl compound metabolic process
S−glycoside biosynthetic process
glucosinolate biosynthetic process
glycosyl compound biosynthetic process

Fig. S4 | Gene modules in *Pst* DC3000 (EV) infected Col-0 samples. (a) Heatmap of DEGs from all cell clusters of EV 3 hpi/5 hpi vs. mock comparisons. DEGs were k-mean clustered into six co-expression modules (Modules EV-1 to EV-6) based on their expression pattern. Columns correspond to the cell clusters; the colored rectangles on top indicate sample types, as indicated on the right, mock (green), EV 3 hpi (yellow) and EV 5 hpi (orange). Heatmap colors reflect the relative expression values (red = high, blue = low), as indicated on the right. (b) Functional enrichment of each module. Dotplots show the top enriched GO terms per module (ClusterProfiler, Benjamini–Hochberg FDR). Dot size represents the number of module genes in the term, and colors encode the adjusted *P* value. EV is short for Pst DC3000 (EV). EV is short for *Pseudomonas syringae* pv. Tomato (*Pst*) DC3000 (EV). DEG, differentially expressed gene; hpi, hours post infection; GO, gene ontology.

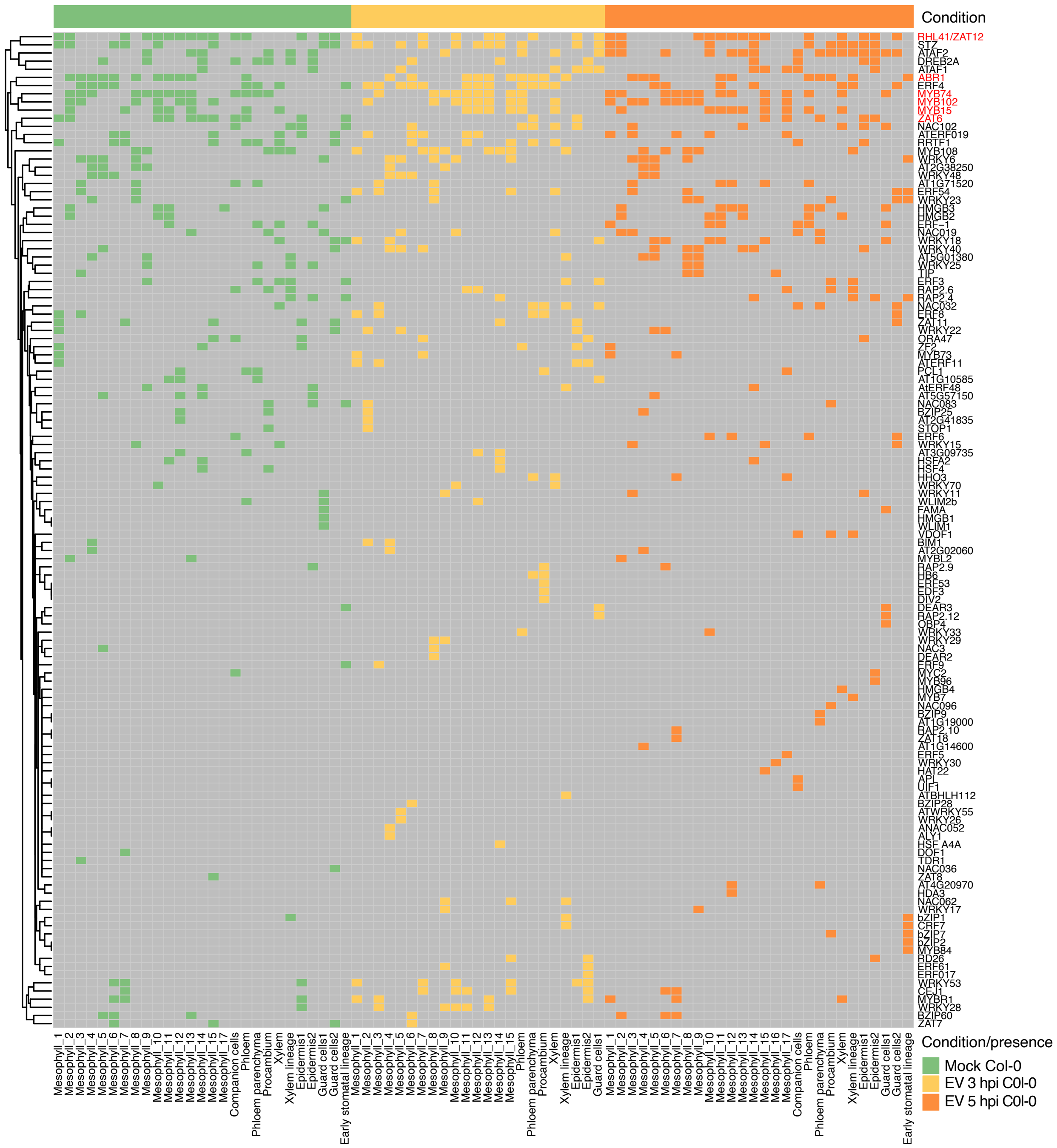

Fig. S5 | Distribution of the top 10 regulons across cell clusters in *Pst* DC3000 (EV) infected Col-0 samples. Presence/absence heatmap summarizing the ten highest-ranking specific regulons detected in each cell cluster. The color bars above indicate the sample types: mock (green), EV 3 hpi (yellow) and EV 5 hpi (orange). Red rectangles highlight transcription factor genes related to stress and growth. EV is short for *Pseudomonas syringae* pv. Tomato (*Pst*) DC3000 (EV). Hpi, hours post infection.

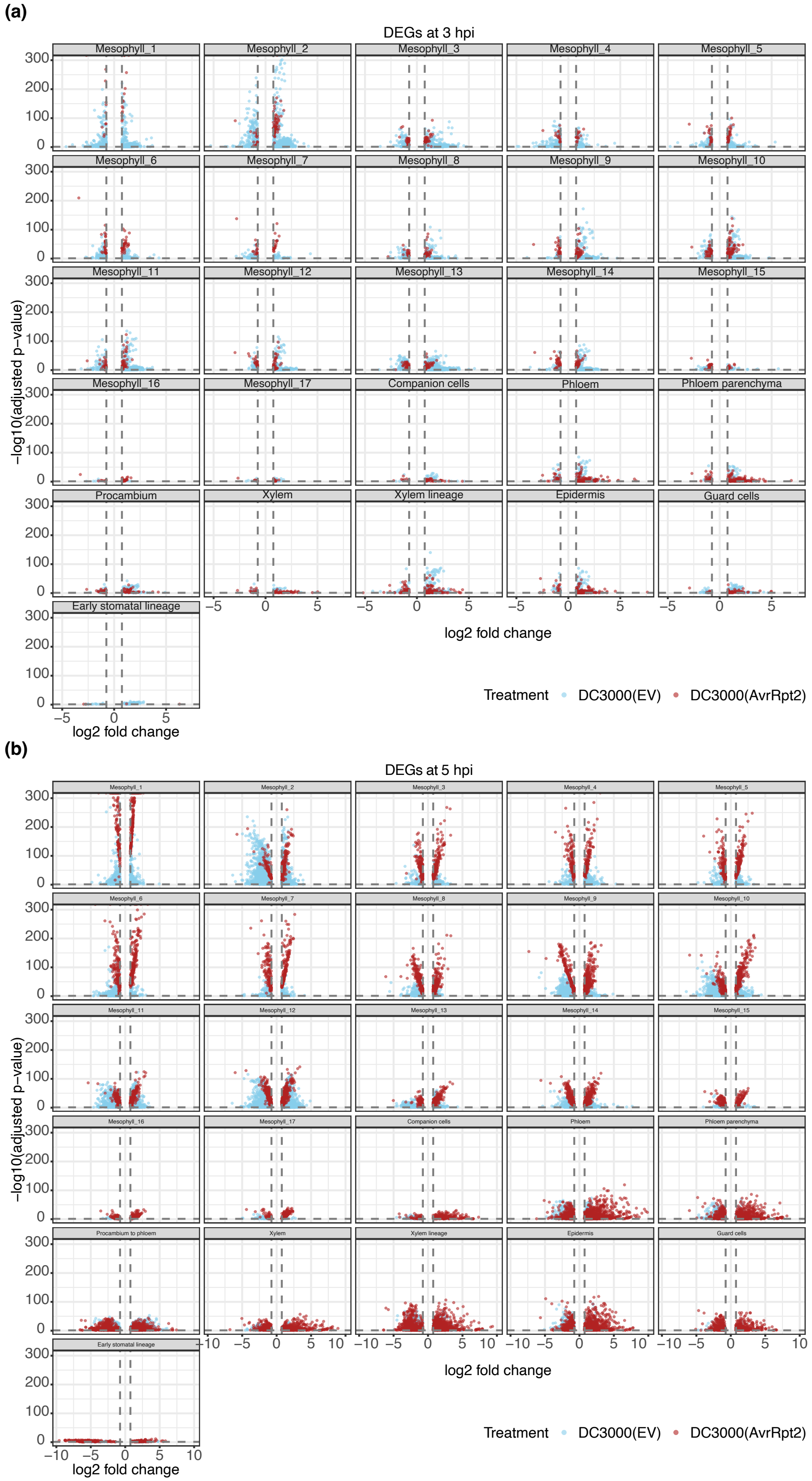
(a)
DEGs at 3 hpi
Mesophyll_1
Mesophyll_2
Mesophyll_3
Mesophyll_4
Mesophyll_5
Mesophyll_6
Mesophyll_7
Mesophyll_8
Mesophyll_9
Mesophyll_10
Mesophyll_11
Mesophyll_12
Mesophyll_13
Mesophyll_14
Mesophyll_15
Mesophyll_16
Mesophyll_17
Companion cells
Phloem
Phloem parenchyma
Procambium
Xylem
Xylem lineage
Epidermis
Guard cells
Early stomatal lineage
−log10(adjusted p−value)
log2 fold change
Treatment DC3000(EV) DC3000(AvrRpt2)
(b)
DEGs at 5 hpi
−log10(adjusted p−value)
log2 fold change
Treatment DC3000(EV) DC3000(AvrRpt2)

Fig. S6 | Distribution of DEGs across cell types in Pst DC3000 (EV) and Pst DC3000 (AvrRpt2) samples. Volcano plots showing the distribution of DEGs in each cell type at 3 hpi (a) and 5 hpi (b) The x-axis indicates the $\log_2$ fold change and the y-axis shows the $-\log_{10}$ adjusted *p*-value. Each point represents one DEG, with blue points corresponding to *Pst* DC3000 (EV) and red points to *Pst* DC3000 (AvrRpt2). EV and A2 are short for *Pseudomonas syringae* pv. Tomato (*Pst*) DC3000 (EV) and *Pst* DC3000 (AvrRpt2). DEG, differentially expressed gene; hpi, hours post infection.

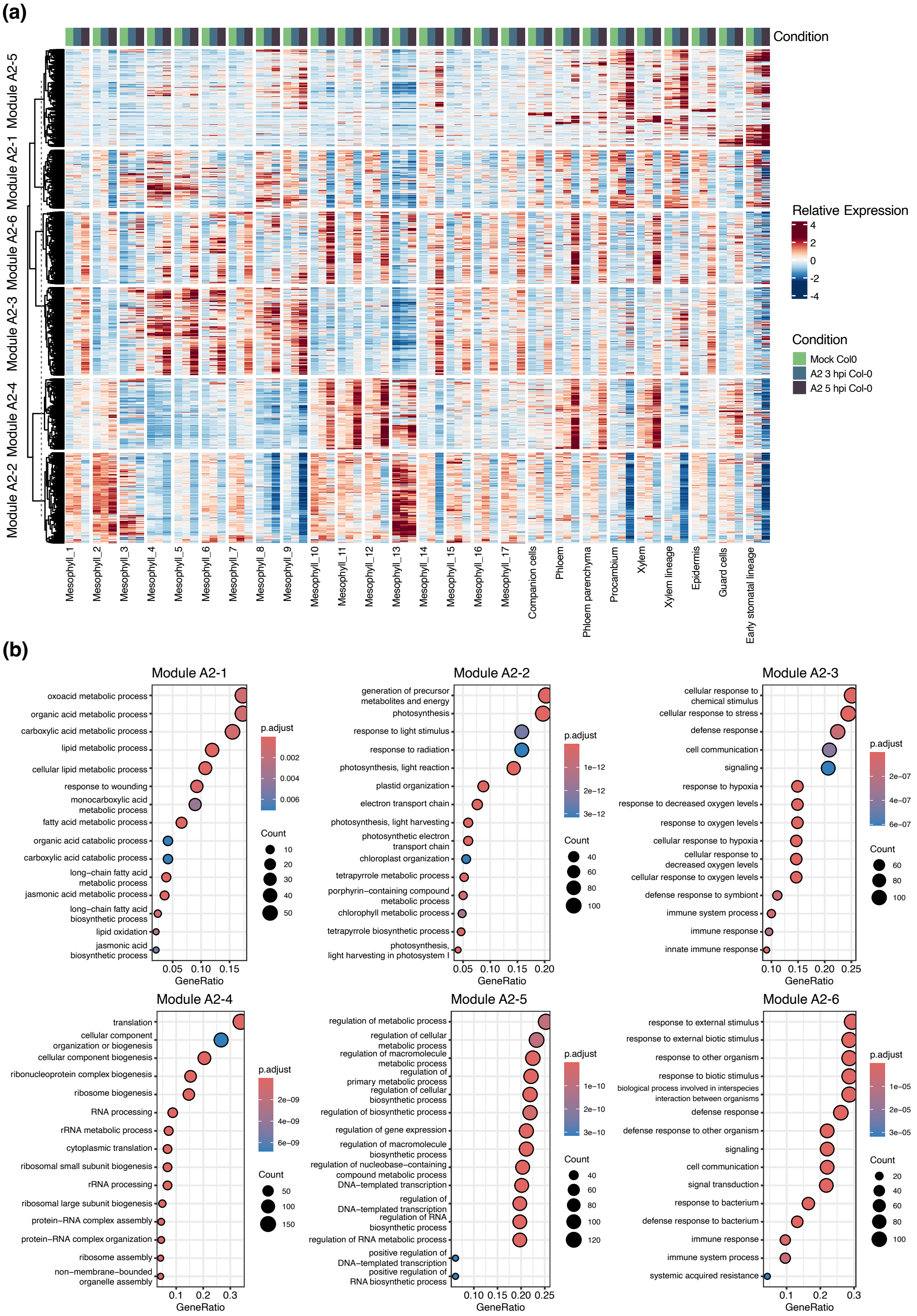
(a)
Condition
Module A2-5
Module A2-1
Module A2-6
Module A2-3
Module A2-4
Module A2-2
Relative Expression
4
2
0
-2
-4
Condition
Mock Col0
A2 3 hpi Col-0
A2 5 hpi Col-0
Mesophyll_1
Mesophyll_2
Mesophyll_3
Mesophyll_4
Mesophyll_5
Mesophyll_6
Mesophyll_7
Mesophyll_8
Mesophyll_9
Mesophyll_10
Mesophyll_11
Mesophyll_12
Mesophyll_13
Mesophyll_14
Mesophyll_15
Mesophyll_16
Mesophyll_17
Companion cells
Phloem
Phloem parenchyma
Procambium
Xylem
Xylem lineage
Epidermis
Guard cells
Early stomatal lineage
(b)
Module A2-1
oxoacid metabolic process
organic acid metabolic process
carboxylic acid metabolic process
lipid metabolic process
cellular lipid metabolic process
response to wounding
monocarboxylic acid metabolic process
fatty acid metabolic process
organic acid catabolic process
carboxylic acid catabolic process
long−chain fatty acid metabolic process
jasmonic acid metabolic process
long−chain fatty acid biosynthetic process
lipid oxidation
jasmonic acid biosynthetic process
GeneRatio
p.adjust
Count
Module A2-2
generation of precursor metabolites and energy
photosynthesis
response to light stimulus
response to radiation
photosynthesis, light reaction
plastid organization
electron transport chain
photosynthesis, light harvesting
photosynthetic electron transport chain
chloroplast organization
tetrapyrrole metabolic process
porphyrin−containing compound metabolic process
chlorophyll metabolic process
tetrapyrrole biosynthetic process
photosynthesis, light harvesting in photosystem I
GeneRatio
p.adjust
Count
Module A2-3
cellular response to chemical stimulus
cellular response to stress
defense response
cell communication
signaling
response to hypoxia
response to decreased oxygen levels
response to oxygen levels
cellular response to hypoxia
cellular response to decreased oxygen levels
cellular response to oxygen levels
defense response to symbiont
immune system process
immune response
innate immune response
GeneRatio
p.adjust
Count
Module A2-4
translation
cellular component organization or biogenesis
cellular component biogenesis
ribonucleoprotein complex biogenesis
ribosome biogenesis
RNA processing
rRNA metabolic process
cytoplasmic translation
ribosomal small subunit biogenesis
rRNA processing
ribosomal large subunit biogenesis
protein−RNA complex assembly
protein−RNA complex organization
ribosome assembly
non−membrane−bounded organelle assembly
GeneRatio
p.adjust
Count
Module A2-5
regulation of metabolic process
regulation of cellular metabolic process
regulation of macromolecule metabolic process
regulation of primary metabolic process
regulation of cellular biosynthetic process
regulation of biosynthetic process
regulation of gene expression
regulation of macromolecule biosynthetic process
regulation of nucleobase−containing compound metabolic process
DNA−templated transcription
regulation of DNA−templated transcription
regulation of RNA biosynthetic process
regulation of RNA metabolic process
positive regulation of DNA−templated transcription
positive regulation of RNA biosynthetic process
GeneRatio
p.adjust
Count
Module A2-6
response to external stimulus
response to external biotic stimulus
response to other organism
response to biotic stimulus
biological process involved in interspecies interaction between organisms
defense response
defense response to other organism
signaling
cell communication
signal transduction
response to bacterium
defense response to bacterium
immune response
immune system process
systemic acquired resistance
GeneRatio
p.adjust
Count

Fig. S7 | Gene modules in *Pst* DC3000 (AvrRpt2) infected Col-0 samples. (a) Heatmap of DEGs from all cell clusters of A2 3 hpi/5 hpi vs. mock. All the DEGs were clustered into six co-expression modules (Modules A2-1 to A2-6) based on their expression pattern (k-mean cluster). Columns correspond to the cell clusters; the color bars above indicate the samples: mock (green), A2 3 hpi (light teal) and A2 5 hpi (dark teal). Colors reflect the relative expression values (red = high, blue = low). (b) Functional enrichment of each module. Dotplots show the top enriched GO terms per module (ClusterProfiler, Benjamini–Hochberg FDR). Dot size represents the number of module genes in the term, and color encodes the adjusted *p* value. A2 is short for *Pst* DC3000 (AvrRpt2). DEG, differentially expressed gene; hpi, hours post infection.

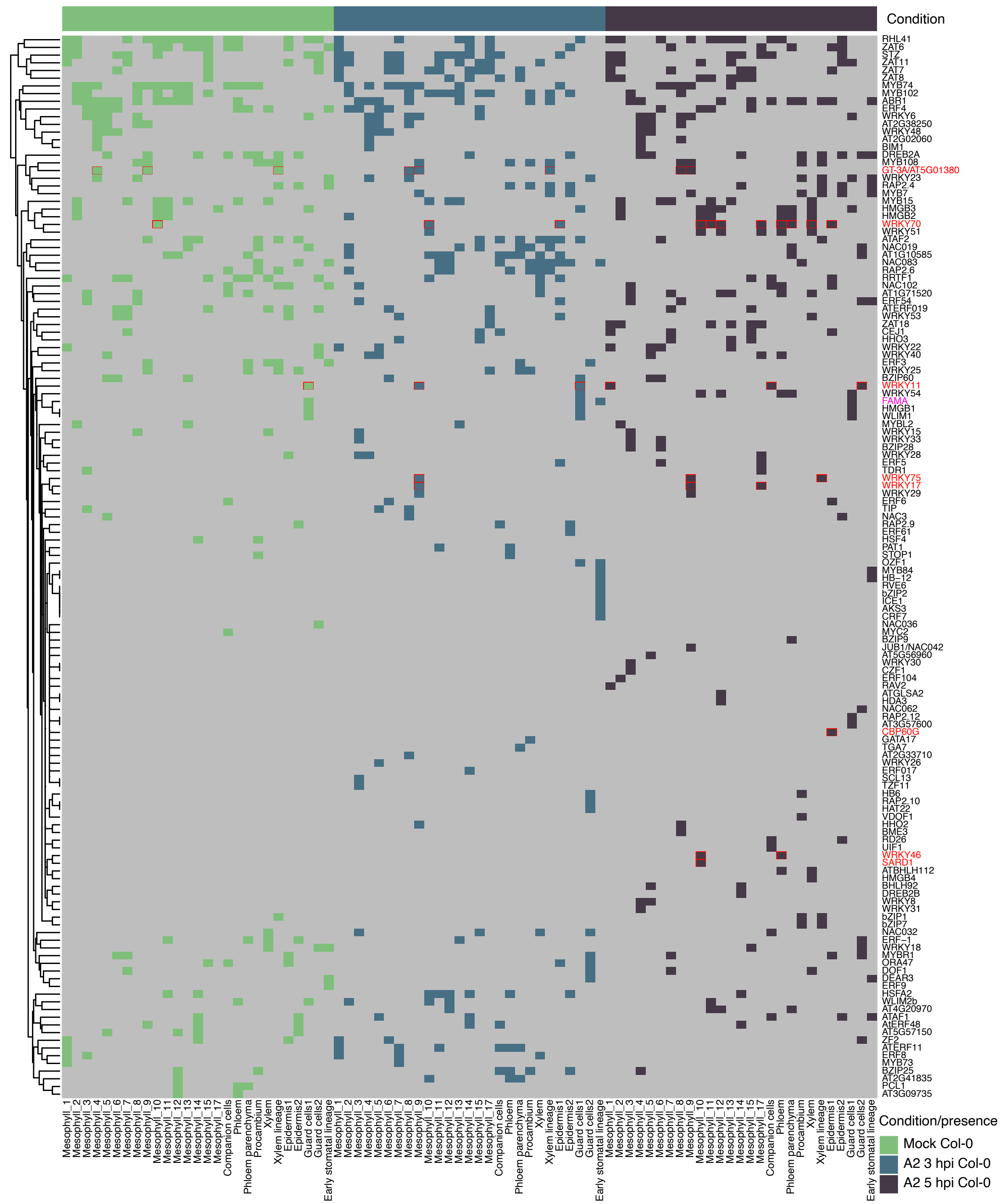

Fig. S8 | Distribution of the top 10 regulons across cell clusters in *Pst* DC3000 (AvrRpt2) infected Col-0 samples. Presence/absence heatmap summarizing the ten highest-ranking specific regulons detected in each cell cluster. The color bars above indicate the sample types: mock (green), A2 3 hpi (light teal) and A2 5 hpi (dark teal). FAMA, GT-3A and SA-related regulons

are highlighted. A2 is short for *Pst* DC3000 (AvrRpt2). SA, salicylic acid; hpi, hours post infection.

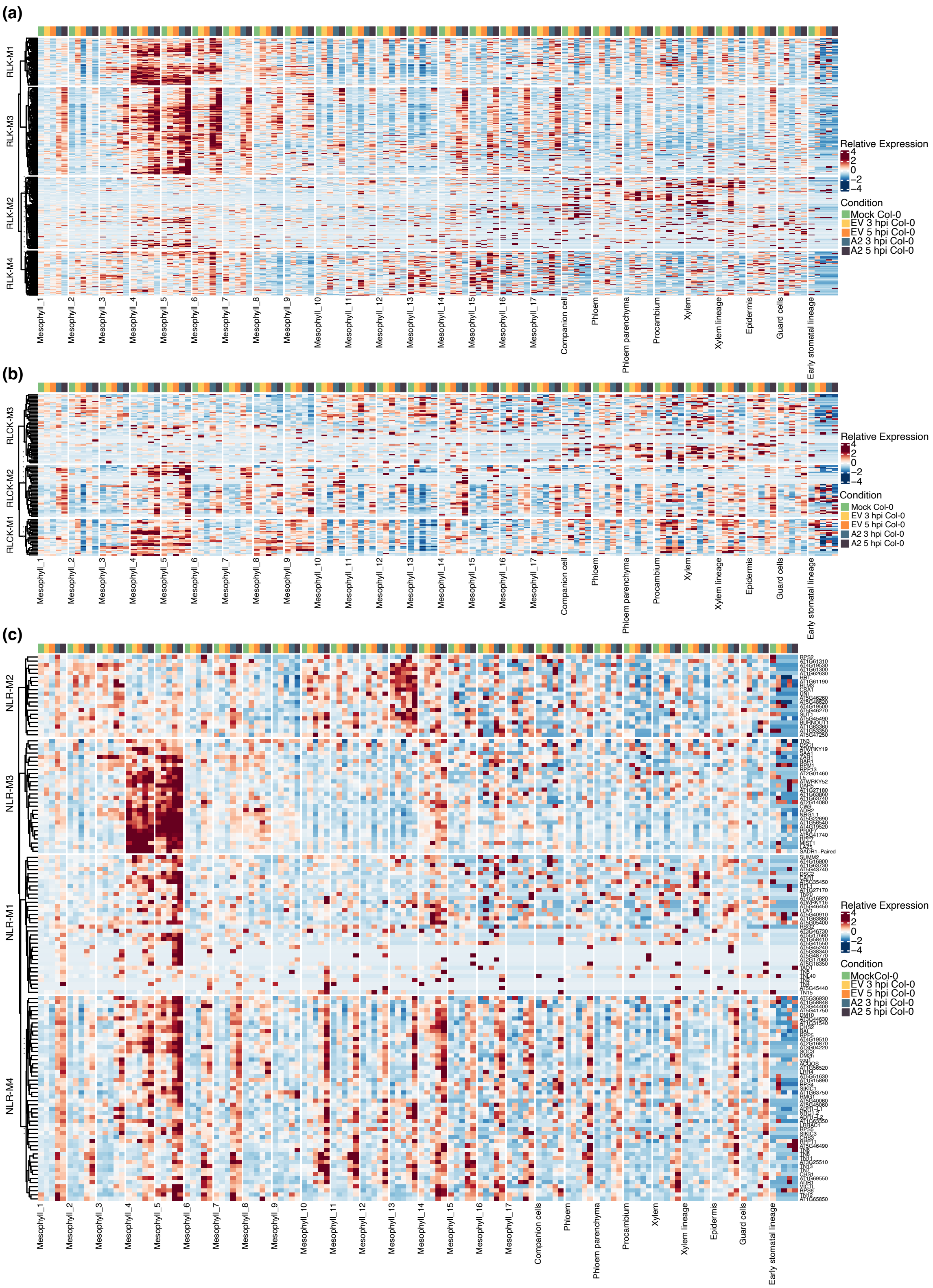
(a)
RLK-M1
RLK-M3
RLK-M2
RLK-M4
Relative Expression
4
2
0
−2
−4
Condition
Mock Col-0
EV 3 hpi Col-0
EV 5 hpi Col-0
A2 3 hpi Col-0
A2 5 hpi Col-0
Mesophyll_1
Mesophyll_2
Mesophyll_3
Mesophyll_4
Mesophyll_5
Mesophyll_6
Mesophyll_7
Mesophyll_8
Mesophyll_9
Mesophyll_10
Mesophyll_11
Mesophyll_12
Mesophyll_13
Mesophyll_14
Mesophyll_15
Mesophyll_16
Mesophyll_17
Companion cell
Phloem
Phloem parenchyma
Procambium
Xylem
Xylem lineage
Epidermis
Guard cells
Early stomatal lineage
(b)
RLCK-M3
RLCK-M2
RLCK-M1
Relative Expression
4
2
0
−2
−4
Condition
Mock Col-0
EV 3 hpi Col-0
EV 5 hpi Col-0
A2 3 hpi Col-0
A2 5 hpi Col-0
Mesophyll_1
Mesophyll_2
Mesophyll_3
Mesophyll_4
Mesophyll_5
Mesophyll_6
Mesophyll_7
Mesophyll_8
Mesophyll_9
Mesophyll_10
Mesophyll_11
Mesophyll_12
Mesophyll_13
Mesophyll_14
Mesophyll_15
Mesophyll_16
Mesophyll_17
Companion cell
Phloem
Phloem parenchyma
Procambium
Xylem
Xylem lineage
Epidermis
Guard cells
Early stomatal lineage
(c)
NLR-M2
NLR-M3
NLR-M1
NLR-M4
Relative Expression
4
2
0
−2
−4
Condition
MockCol-0
EV 3 hpi Col-0
EV 5 hpi Col-0
A2 3 hpi Col-0
A2 5 hpi Col-0
Mesophyll_1
Mesophyll_2
Mesophyll_3
Mesophyll_4
Mesophyll_5
Mesophyll_6
Mesophyll_7
Mesophyll_8
Mesophyll_9
Mesophyll_10
Mesophyll_11
Mesophyll_12
Mesophyll_13
Mesophyll_14
Mesophyll_15
Mesophyll_16
Mesophyll_17
Companion cells
Phloem
Phloem parenchyma
Procambium
Xylem
Xylem lineage
Epidermis
Guard cells
Early stomatal lineage

Fig. S9 | Expression patterns of RLKs, RLCKs and NLRs in *Pst* DC3000 (EV) and *Pst* DC3000(AvrRpt2) infected Col-0 samples. Heatmap of RLK (a), RLCK (b) and NLR genes (c) from all cell clusters of Mock, EV 3 hpi/5 hpi and A2 3 hpi/5 hpi samples. RLK, RLCK and NLR genes are clustered based on their expression patterns (k-mean cluster). Columns correspond to the cell clusters; the color bars above indicate the sample types: mock (green), EV 3 hpi (yellow), EV 5 hpi (orange), A2 3 hpi (light teal) and A2 5 hpi (dark teal). Color shows the relative expression value (red = high, blue = low). Genes in each module and the relative expression levels are listed in Table S14-16). EV and A2 are short for *Pseudomonas syringae* pv. Tomato (*Pst*) DC3000 (EV) and *Pst* DC3000 (AvrRpt2). DEG, differentially expressed gene; hpi, hours post infection.

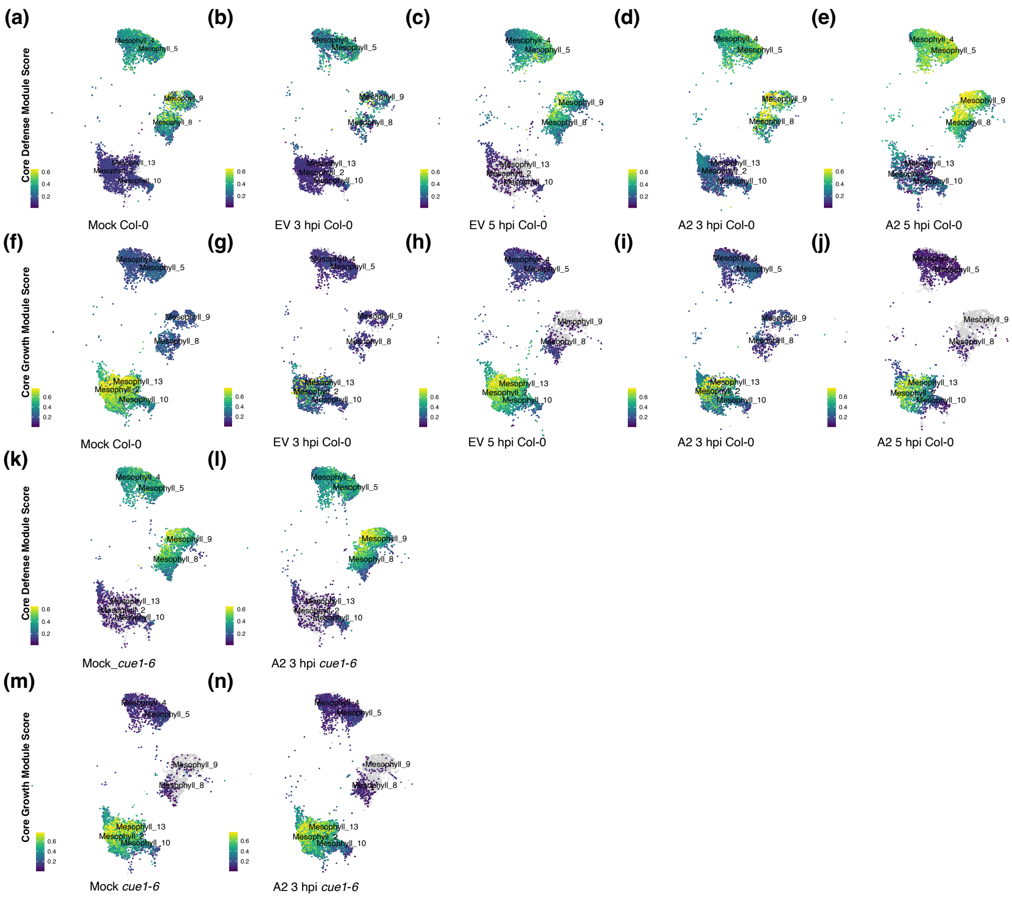

Fig. S10 | Expression of core defense and growth module genes in mock and *Pst* DC3000 (EV) or *Pst* DC3000 (AvrRpt2) infected Col-0 and *cue1*-6 samples. UMAPs showing average module scores of core defense and core growth genes (defined in Fig. 5a) across defense-over-growth (Mesophyll_4, _5, _8 and _9) and growth-over-defense (Mesophyll_2, _10 and _13) cell clusters. Yellow indicates higher and purple lower module scores. UMAP, uniform manifold approximation and projection.

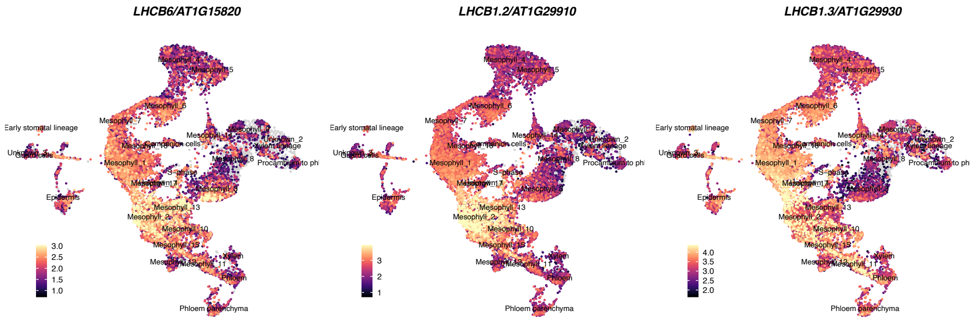

Fig. S11 | Expression pattern of photosynthesis-related genes. UMAPs of the mock Col-0 atlas colored by scaled expression levels of *LHCB6*, *LHCB1.2*, and *LHCB1.3*. Yellow marks cells with higher transcript abundance, purple marks lower abundance. UMAP, uniform manifold approximation and projection.

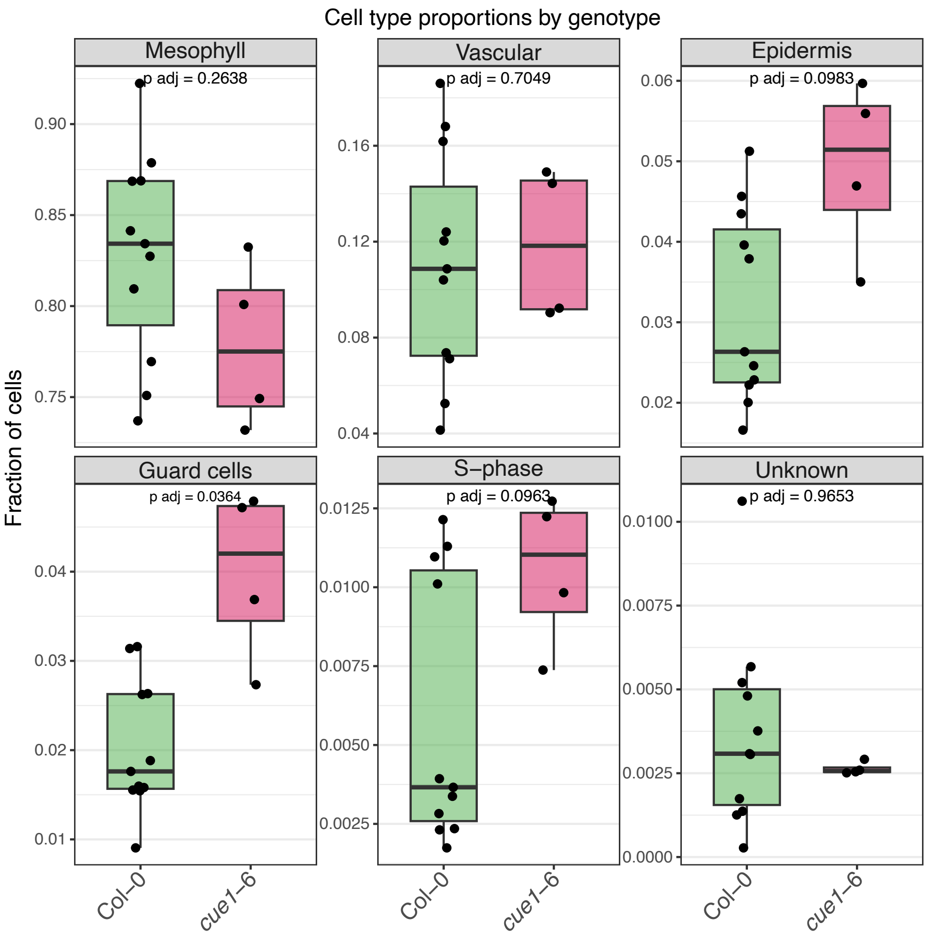

Fig. S12 | Cell-type proportions in Col-0 versus *cue1*-6. Box plots show the fraction of every major cell type, each dot representing an individual library with Col-0 (n = 11) and *cue1*-6 (n = 4). Benjamini–Hochberg FDR for the moderated *t*-test was performed and the adjusted *p* value is labeled.

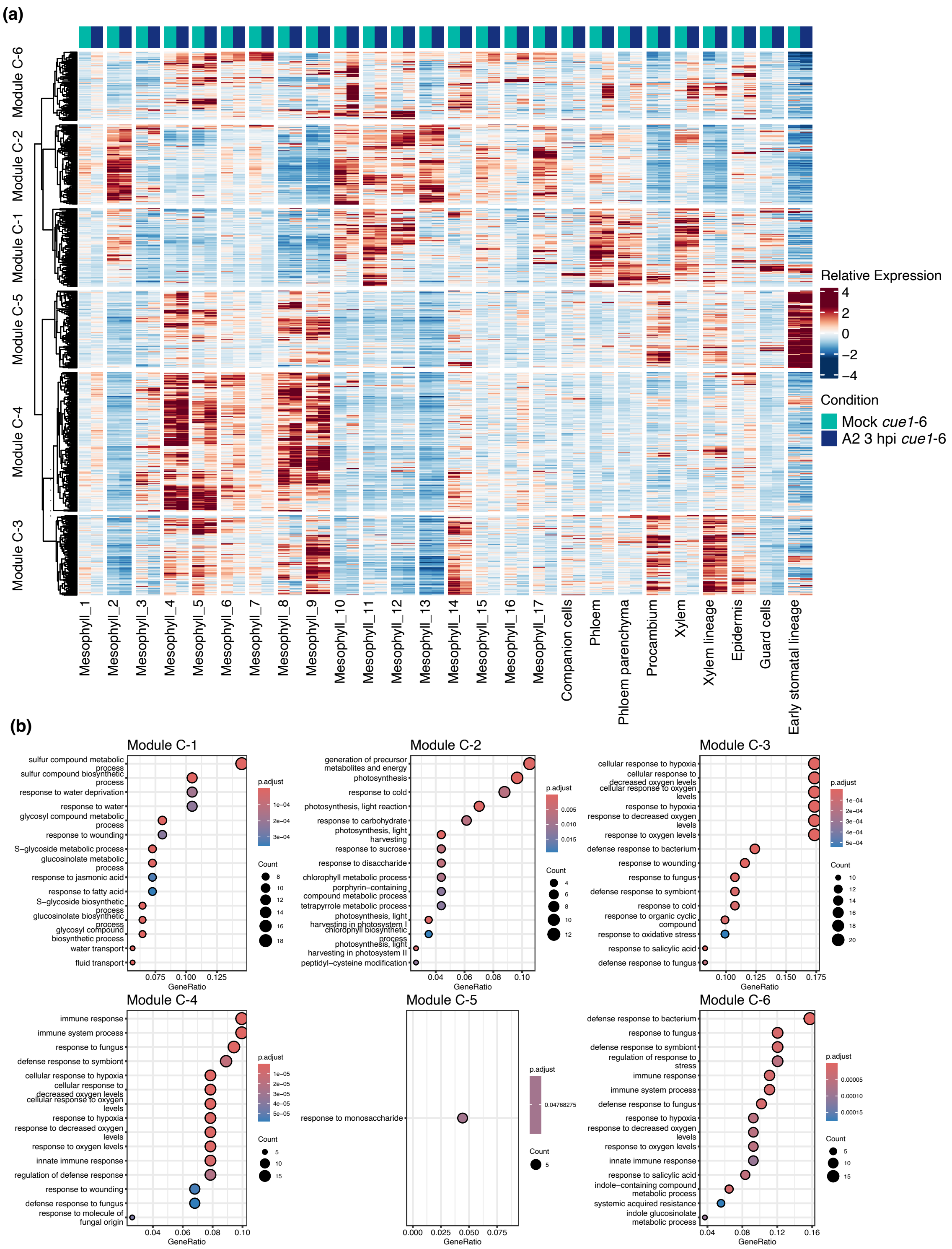

Fig. S13 | Gene modules in *Pst* DC3000 (AvrRpt2) infected *cue1*-6 samples (a) Heatmap of DEGs from all cell clusters of A2 3 hpi vs. mock in *cue1*-6. All the DEGs were clustered into six co-expression modules (Modules *cue1* (C)-1 to C-6) based on their expression pattern (k-mean

cluster). Columns correspond to the cell clusters; the color bars above indicate the sample types: *cue1*-6 mock (green) and *cue1*-6 A2 3 hpi (blue). Color shows the relative expression value (red = high, blue = low). (b) Functional enrichment of each module. Dotplots show the top enriched GO terms per module (ClusterProfiler, Benjamini–Hochberg FDR). Dot size represents the number of module genes in the term, and color encodes the adjusted *p* value. A2 is short for *Pseudomonas syringae* pv. Tomato (*Pst*) DC3000 (AvrRpt2). DEG, differentially expressed gene; hpi, hours post infection; GO, gene ontology.

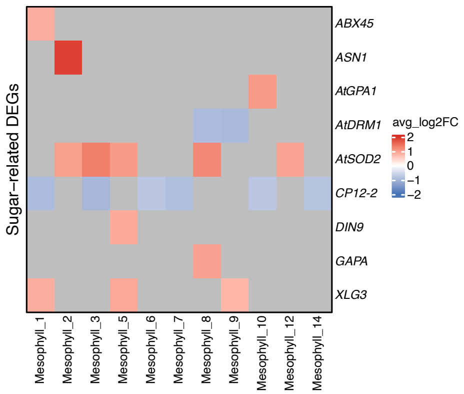

Fig. S14 | Sugar-related DEGs from *Pst* DC3000 (AvrRpt2) infected samples at 3 hpi in *cue1*-6. Heatmap showing the log_2 fold change of sugar-related DEGs in *Pst* DC3000 (AvrRpt2) infected *cue1*-6 samples compared with mock samples at 3 hpi. Red indicates upregulation and blue indicates downregulation. DEGs, differentially expressed gene; hpi, hours post infection.

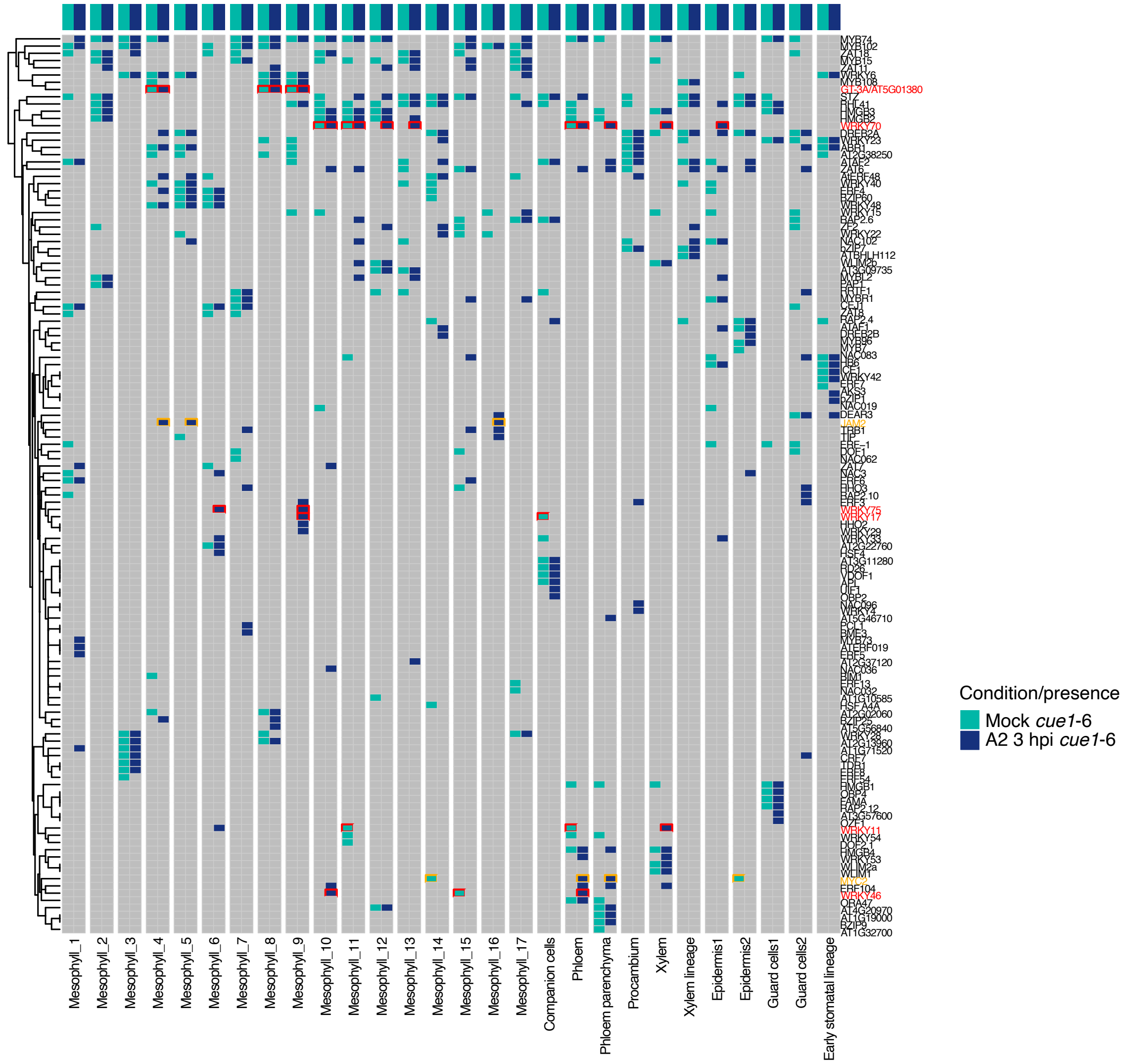

Fig. S15 | Distribution of the top 10 regulons across cell clusters in *Pst* DC3000 (AvrRpt2) infected *cue1*-6 samples. Presence/absence heatmap summarizing the ten highest-ranking specific regulons detected in each cell cluster. The color bars above indicate the sample types: *cue1*-6 mock (green) and *cue1*-6 A2 3 hpi (blue). GT-3A and SA-related regulons are highlighted in red, and a JA-related regulon is highlighted in yellow. A2 is short for *Pseudomonas syringae* pv. Tomato (*Pst*) DC3000 (AvrRpt2). SA, salicylic acid, JA, jasmonic acid; hpi, hours post infection.

Reference for Table S2

Kim J-Y, Symeonidi E, Pang TY, Denyer T, Weidauer D, Bezrutczyk M, Miras M, Zöllner N, Hartwig T, Wudick MM, *et al.* 2021. Distinct identities of leaf phloem cells revealed by single cell transcriptomics. *The Plant cell* **33**: 511–530.

Lopez-Anido CB, Vatén A, Smoot NK, Sharma N, Guo V, Gong Y, Anleu Gil MX, Weimer AK, Bergmann DC. **2021**. Single-cell resolution of lineage trajectories in the Arabidopsis stomatal lineage and developing leaf. *Developmental cell* **56**: 1043–1055.e4.

Tenorio Berrío R, Verstaen K, Vandamme N, Pevernagie J, Achon I, Van Duyse J, Van Isterdael G, Saeys Y, De Veylder L, Inzé D, *et al.* 2022. Single-cell transcriptomics sheds light on the identity and metabolism of developing leaf cells. *Plant physiology* **188**: 898–918.
